# Ang-(1-7)/ MAS1 receptor axis inhibits allergic airway inflammation via blockade of Src-mediated EGFR transactivation in a murine model of asthma

**DOI:** 10.1371/journal.pone.0224163

**Published:** 2019-11-01

**Authors:** Ahmed Z. El-Hashim, Maitham A. Khajah, Rhema S. Babyson, Waleed M. Renno, Charles I. Ezeamuzie, Ibrahim F. Benter, Saghir Akhtar

**Affiliations:** 1 Department of Pharmacology & Therapeutics, Faculty of Pharmacy, Kuwait University, Kuwait City, Kuwait; 2 Department of Anatomy, Faculty of Medicine, Kuwait University, Kuwait City, Kuwait; 3 Department of Pharmacology and Toxicology, Faculty of Medicine, Kuwait University, Kuwait City, Kuwait; 4 Faculty of Medicine, Eastern Mediterranean University, Famagusta, North Cyprus; 5 College of Medicine, Qatar University, Doha, Qatar; Max Delbruck Centrum fur Molekulare Medizin Berlin Buch, GERMANY

## Abstract

The angiotensin-(1–7) [Ang-(1–7)]/MAS1 receptor signaling axis is a key endogenous anti-inflammatory signaling pathway. However, the mechanisms by which its mediates the anti-inflammatory effects are not completely understood. Using an allergic murine model of asthma, we investigated whether Ang-1(1–7)/MAS1 receptor axis a): inhibits allergic inflammation via modulation of Src-dependent transactivation of the epidermal growth factor receptor (EGFR) and downstream signaling effectors such as ERK1/2, and b): directly inhibits neutrophil and/or eosinophil chemotaxis *ex vivo*. Ovalbumin (OVA)-induced allergic inflammation resulted in increased phosphorylation of Src kinase, EGFR, and ERK1/2. In addition, OVA challenge increased airway cellular influx, perivascular and peribronchial inflammation, fibrosis, goblet cell hyper/metaplasia and airway hyperresponsiveness (AHR). Treatment with Ang-(1–7) inhibited phosphorylation of Src kinase, EGFR, ERK1/2, the cellular and histopathological changes and AHR. Ang-(1–7) treatment also inhibited neutrophil and eosinophil chemotaxis *ex vivo*. These changes were reversed following pre-treatment with A779. These data show that the anti-inflammatory actions of Ang-(1–7)/ MAS1 receptor axis are mediated, at least in part, via inhibition of Src-dependent transactivation of EGFR and downstream signaling molecules such as ERK1/2. This study therefore shows that inhibition of the Src/EGRF/ERK1/2 dependent signaling pathway is one of the mechanisms by which the Ang-(1–7)/ MAS1 receptor axis mediates it anti-inflammatory effects in diseases such as asthma.

## Introduction

Chronic airway inflammatory response is characterized by repeating cycles of allergen insults and airway repair leading to structural and functional changes such as airway remodeling, airway obstruction and hyperresponsiveness (AHR) [1, 2]. The epithelial cell in particular, with its unique position, has been shown to play a critical role in driving these deleterious changes in the asthmatic airway through the generation of many pro-inflammatory signals, such as through activation of epidermal growth factor receptor (EGFR) and its downstream signaling molecules [3, 4].

Over the last two decades, many cytokines/chemokines have been shown to have important regulatory and effector roles in asthma pathogenesis [5]. Nonetheless antagonism of their signaling pathways has not resulted in the anticipated therapeutic benefits in the wider asthma population with only modest effects seen in severe asthma [6, 7]. Interestingly, as predicted by “network biology analysis”, to have any significant impact on disease metrics, several “nodes” need to be targeted simultaneously, rather than blockade of individual mediator signaling pathways [8]. This approach is in line with the mechanisms of action of steroids, the most effective asthma therapy, which act to “switch off” many pro-inflammatory signaling pathways simultaneously and is so far the gold standard in asthma therapy [9].

There is a growing body of evidence suggesting an important and beneficial role for the newly discovered arm of the renin-angiotensin-aldosterone system (RAAS); angiotensin converting enzyme 2 (ACE2)/angiotensin (Ang) 1-7/ MAS1 receptor axis in the pathogenesis of various disorders [10, 11]. Indeed, Ang-(1–7) was shown to oppose various effector functions of Ang II in regulating cardiovascular and renal functions [10, 12–15]. Also, anti-inflammatory actions of Ang-(1–7) have been demonstrated in numerous inflammatory models such as arthritis [16], atherosclerosis plaque [17], and more recently in a model of colitis [18]. We, and others, have reported that treatment with the Ang-(1–7) or the non-peptide compound MAS1 receptor agonist AVE 0991 (AVE) ameliorates OVA-induced airway perivascular and peribronchial inflammation, fibrosis, goblet cell hyper/metaplasia, and inflammatory cell counts in the bronchoalveolar lavage fluid (BALF) [19, 20] in a model of allergic inflammation. The important role that (Ang) 1–7 MAS1/receptor axis plays in regulating inflammation has also been highlighted in a study showing that deletion of the MAS1 genes enhances the airway inflammatory response to allergen challenge [21]. Altogether, these studies underscore the important role that Ang-(1–7)/MAS1 receptor axis plays in counteracting pro-inflammatory pathways.

The exact molecular mechanism of action of Ang-(1–7)/MAS1 axis is not known but it has been shown to modulate various arms of the inflammatory response such as inhibition of cytokines such as TNF-α, IL-1β, CXCL1 [16, 22] and activity of pro-inflammatory enzymes such as NADPH oxidase (NOX) [23]. More recently, using a Th1 driven colitis model, we have shown that Ang-(1–7)/MAS1 axis modulates inflammatory cell functions such as induction of neutrophil apoptosis, and inhibition of chemotaxis and superoxide release *in vitro* [24]. However, the effects of activation of the Ang-(1–7)/MAS1 receptor axis on neutrophil and/or eosinophil chemotaxis, within an allergic inflammation response, is not known.

The epithelial growth factor (EGF) and its receptor have been implicated in the pathogenesis of diseases such as cancers, diabetes and asthma [25–32]. In asthma for example, enhanced expression of EGF/EGFR was observed in the bronchial epithelium, airway glands, smooth muscle and basement membrane of asthmatic individuals, and correlated well with sub-epithelial basement membrane thickening [25]. In animal models of asthma, selective EGFR inhibitors such as AG1478 and gefitinib, significantly reduced airway smooth muscle hyperplasia/remodeling, eosinophil recruitment, inflammation, AHR and epithelial and goblet cell proliferation [27, 33, 34]. Members of the Src family of tyrosine kinases have also been directly implicated in a number of signaling pathways involved in asthma [35–38] and allergen activation of IgE receptors [39–41]. We have recently shown, in both asthma and diabetes disease models, in which EGFR activity is enhanced, that inhibition of Src kinase reduces EGFR transactivation and ameliorates the disease features [4, 42, 43].

The objective of this study was to investigate, using an allergic murine model of asthma, whether the Ang-1(1–7)/MAS1 receptor axis a) inhibits allergic inflammation via modulation of Src kinase and/or EGFR and/or their downstream signaling pathways through ERK1/2 and b), modulates neutrophil and/or eosinophil chemotaxis *ex vivo* to bronchoalveolar lavage (BALF) from OVA challenged mice.

## Methods

### Animals

Male BALB/c mice (6–8 weeks old) used in this study were maintained under temperature-controlled conditions with an artificial 12 h light/dark cycle and were allowed standard chow and water *ad libitum*. All studies involving animals are reported in accordance with the ARRIVE guidelines for reporting experiments involving animals. All experimental protocols were approved by the Animal Welfare and Use of Laboratory Animals Committee in the Health Sciences Center, Kuwait University and complied with the ARRIVE Guidelines and were carried out in accordance with the EU Directive 2010/63/EU for animal experiments and the National Institutes of Health guide for the care and use of Laboratory animals (NIH Publications No. 8023, revised 1978).

### Immunization and intranasal challenge and drug treatment protocols

BALB/c mice were immunized once by intraperitoneal (i.p.) injection of 10 μg ovalbumin (OVA) in 0.2 ml of alu-Gel-S (Alu-Gel-S; SERVA Electrophoresis GmbH) on day 0. Ten days later, the animals were challenged intranasally once a day over 4 consecutive days with 30 μg OVA dissolved in 50 μl PBS solution. Control animals were similarly immunized with OVA but challenged intranasally with 50 μl PBS. All intranasal administrations were done following light anesthesia with halothane.

Five treatment groups (n = 9–14) were established. Groups A and B were treated (i.p.) with the vehicle (water) for A779 and then 1 h later treated with the vehicle (water) for Ang-(1–7) and 30 minutes thereafter, mice were challenged intranasally with PBS and OVA respectively. One hour subsequently, these mice were treated with the vehicle (water) for Ang-(1–7). Group C was treated (i.p.) with the vehicle (water) for A779 and 1 h later treated with Ang-(1–7) (0.3 mg/kg; i.p.). Thirty minutes thereafter, these mice were challenged intranasally with OVA and 1 h subsequently were treated with Ang-(1–7) (0.3 mg/kg; i.p.). Group D was treated with A779 (1 mg/kg; i.p.), and 1 h later, the mice were treated with Ang-(1–7) (0.3 mg/kg; i.p.). Thirty minutes thereafter, mice were challenged intranasally with OVA, and 1 h later they were treated with Ang-(1–7) (0.3 mg/kg; i.p.). Group E was treated (i.p.) with the vehicle (water) and 1 h later they treated with dexamethasone (1 mg/kg; i.p.). Thirty minutes thereafter, mice were challenged intranasally with OVA. The drug/vehicle treatment and PBS/ovalbumin intranasal challenges were continued for four consecutive days.

### BAL fluid cell counts and lung histology

BAL fluid was collected by cannulating the trachea and washing the lungs with saline solution (4 × 0.3 ml each) after sacrificing the animals with an over dose with halothane. BAL cells were counted using a particle-size counter (Z1 Single Threshold; Beckman Coulter) and cytosmears were prepared for differential count. Cells were stained with Diff-Quik and a differential count of 200 cells was performed using standard morphologic criteria. Results are expressed as total cell count/ml and as total macrophages, lymphocytes, neutrophils, and eosinophils/ml in BAL fluid.

For histology, pieces of lung tissue were removed and fixed in 10% buffered formalin, embedded in paraffin wax and sectioned into 5-μm-thick slices. The sections were processed and stained separately with H&E stain, Masson's Trichrome stains and periodic acid–Schiff (PAS) according to standard methods. Sections were examined under light microscope and the severity of pathologic changes scored independently by two experienced histologists unfamiliar with the slides. Score coding was as follows: (1 = normal, 2 = mild, 3 = moderate, 4 = severe and 5 = highly severe).

### Measurement of airway responsiveness

Measurement of airway responsiveness was done on a separate set of animals 24 h after last OVA or PBS challenge using a Buxco FinePointe series RC site (DSI, Wilmington, NC), according to the manufacturer’s guidelines. In short, mice were anesthetized with an intraperitoneal injection of ketamine/xylazine (1:0.1 mg/kg) cocktail and tracheotomized with a steel 18-gauge cannula. Mice were subsequently mechanically ventilated at a rate of 150 breaths/min, and tidal volume of 0.15 ml, using a computerized small animal ventilator (FinePointe site), as previously described [42, 44, 45]. After 5 min of stabilization followed, by administration of PBS, airway resistance was measured by exposing mice to aerosolized methalcholine (6.25–50.0 mg/ml, 5 μl per delivery) delivered by an aerogen nebulizer administration, and reported as total lung resistance (*R*_L_) (centimeters H_2_O per milliliter per second).

### Immunofluorescence

Lung tissues were processed as described above. Immunofluorescence was performed as previously described [46]. In brief, lung sections were incubated in blocking solution (5% bovine serum albumin (BSA) + 0.3% Triton X-100 in PBS) for 1 h, followed by incubation overnight at 4°C with primary antibodies [p-EGFR, p-Src, and p-ERK1/2 (1:50–1:100 dilution) or only 1% BSA (for negative control); Cell Signaling, USA], diluted in 1% blocking solution. On the following day, sections were washed and incubated with secondary antibody conjugated to Alexa Fluor 555 (Goat anti rabbit SFX kit; Life Technologies, USA, 1:400 dilution) for 2 h at room temperature) in the dark. After several washes in PBS, sections were stained with 4’, 6 diamidino-2- phenyl indole and mounted. Images were captured on a ZIESS LSM 700 confocal microscope and fluorescence intensity estimated in defined fields using Image J software package. The laser setting and photo processing were equal amongst the different treatment groups for each protein. 40x magnification for the tested molecules were equally modified in terms of sharpness and contrast to show localization of the phospho proteins in the lung tissue.

### Western blotting

The right lobes from the dissected lungs of the mice were snap-frozen in liquid nitrogen and stored at −80°C. The tissue samples were defrosted in ice then transferred to lysis buffer (pH 7.6) containing 50 mM Tris-base, 5mM EGTA, 150mM NaCl, 1% Triton 100, 2mM Na_3_VO_4_, 50mM NAF, 1mM PMSF, 20 μM phenyl arsine, 10 mM sodium molybdate,

10 μgmL^–1^ leupeptin and 8 μgmL^–1^ aprotinin. Using homogenizer, the tissues were homogenized for 10 second, 3 times. The samples were left to lyse completely by incubation on ice for 30 min. Lysates were then centrifuged at 13000 rpm for 10 min at 4°C and supernatants were collected and protein concentration estimated by Bio-Rad Bradford protein assay (Bio-Rad, Hercules, CA, USA). Actin was used as a loading control. The following antibodies from Cell Signaling (USA) were used in this study: p-EGFR-Antibody (Y1068) (rabbit; Cat. No. 2234L), pp44/42 MAPK (ERK1/2) (137F5) (Rabbit; Cat. No. 4695T), and from pSrc Family (Tyr416) (Cat.No.6943S), whereas the anti-actin rabbit polyclonal IgG (1 μl/10 ml) (Cat. No. A-2066) was obtained from Sigma Chemical Co, USA. Aliquots containing equal amounts of protein were subjected to SDS-PAGE and transferred electrophoretic ally onto nitrocellulose membrane (Schleicher & Schuell, Dassel, Germany). The membranes were blocked with 5% bovine serum albumin (BSA) and then incubated with primary antibodies (1:1000 in 5% BSA) or β-actin primary antibodies at 4 °C overnight. Membranes were incubated with either monoclonal antibodies (Cell Signaling, Danvers, MA, USA) to detect phosphorylated forms of EGF receptor (bands seen at approximately 175kDa), ERK1/2 (at 42/44kDa), pSrc (at 56-62kDa), and subsequently with appropriate secondary antibodies conjugated to horseradish peroxidase (Amersham, Buckinghamshire, UK).

To ensure equal loading of proteins, β-actin levels were detected using primary rabbit anti-human β-actin antibody (1:1000 in 5% BSA) followed by the secondary anti-rabbit IgG horse-radish peroxidase conjugated antibody (Cell Signaling). Immunoreactive bands were detected with Super Signal chemiluminescent substrate (Immuno Cruz Western blotting luminal reagent SC-20428, Santa Cruz Biotechnology) using Kodak autoradiography film (Care stream Biomax Xarfil 1660760). Images were finally analyzed and all data were normalized to β-actin levels. The experiment was run twice with lung samples from three different mice, in each treatment groups (pooled), in each run.

### Isolation of murine bone-marrow derived neutrophils

Neutrophils were isolated from murine tibial and femoral bone marrow as described previously [24]. Briefly, mice were euthanized and the femurs and tibias dissected from the animal and the ends of bones removed. The marrow was flushed from the bone with ice-cold 50 ml PBS and then centrifuged at 1300 rpm for 6 min at 4°C. After harvesting of bone-marrow-derived cells by flushing with PBS, the cells were re-suspended in 3 ml of 52% Percoll and layered on a 3-step Percoll gradient (72%, 64%, and 52% plus cells), and centrifuged (2600 rpm for 30 min at 4 °C). Purified neutrophils were removed from the layer between the 64% and 72% Percoll and washed once with ice-cold PBS and suspended in RPMI culture media containing 20% FBS at a concentration of 10^7^cells/ml. Neutrophil viability was >95% based on Trypan blue exclusion test.

### Assessment of neutrophil chemotaxis (under-agarose assay) *in vitro*

The under agarose chemotaxis assay [24, 46] was used to determine the effect of BALF on cell chemotaxis. Tissue culture dishes were filled with 3 ml of 0.5% agarose solution. After solidification, three wells (3.5 mm diameter) were created in the gel 2.5 mm apart in a horizontal line. The center well was loaded with 10 μl of BALF taken from vehicle- (control) or OVA-treated mice, and the outer wells were loaded with 10 μl neutrophils (10^7^ cells/ml) (pretreated for 30 min with vehicle or different concentrations of Ang- (1–7) and incubated for 4 h (at 37 °C, 5% CO_2_). Results were analyzed by visual microscopic examination (×100). The degree of chemotaxis was determined by counting the number of cells which migrate towards the source of chemoattractant minus the number migrating away from it.

### Isolation of human blood eosinophils

Fresh blood was obtained from healthy individuals, after getting their informed consent, with no history of allergic disease nor had taken any medication in the last 72 h. The methods and protocol for these experiments were performed in accordance to and approved by the “Ethical Committee of the Faculty of Medicine, Kuwait University”. Granulocytes were isolated from heparinized (10 IU/ml) blood by erythrocyte sedimentation, followed by percoll gradient centrifugation as previously described [44]. Eosinophils were separated using negative selection with the immunomagnetic method as previously described [47]. The eosinophil purity was assessed by differential count of a Wright-Giemsa stained cytosmear and was routinely >98%. Viability was determined by Trypan blue exclusion and exceeded 98%.

### Boyden chamber assay for eosinophil chemotaxis

Peripheral blood derived eosinophils were used for chemotaxis assay using the Boyden chamber as previously described [48]. Purified eosinophils (2 × 10^5^) (pretreated for 30 min with vehicle or different concentrations of Ang -(17); A779 pretreated cells were pretreated for 30 min prior to Ang -(1–7). Cell were then placed in the upper wells and in the lower wells, 500 μl of BALF derived from mice challenged with PBS (vehicle) or OVA pretreated and allowed to migrate for 1 h (37 °C/5%CO_2_). The transmigrated cells were determined by counting under the microscope by using a hemocytometer.

### Statistical analyses

All numerical values were expressed as means + S.E.M. Total cell counts represent the number of BALF cells/ml. Differential cell counts represent the absolute number of each cell type/ml of BALF. Absolute R_L_ values were calculated and used as an index of the airway responsiveness to methacholine. For the histopathology, a semi-quantitative 5-level lung pathology score was used to grade the extent of abnormalities in each microscopic field at 200X. All data were initially assessed for normality. For the airway responsiveness, a two-way repeated measure analysis of variance followed by a Bonferroni post hoc test was used. One-way analysis of variance (ANOVA) test followed by Bonferroni post hoc was used to compare differences between individual groups for both total and differential cell count and histopathological data. An ANOVA test followed by Bonferroni post hoc test was used for the immunofluorescence data. The mean difference was considered as significant at a probability level of less than 0.05. All results analysis was performed using GraphPad Prism.

## Results

### Effect of Ang-(1–7) on phosphorylation of Src, EGFR and ERK1/2 as determined by immunofluorescence

#### Effect on Src kinase

**Fig 1:**
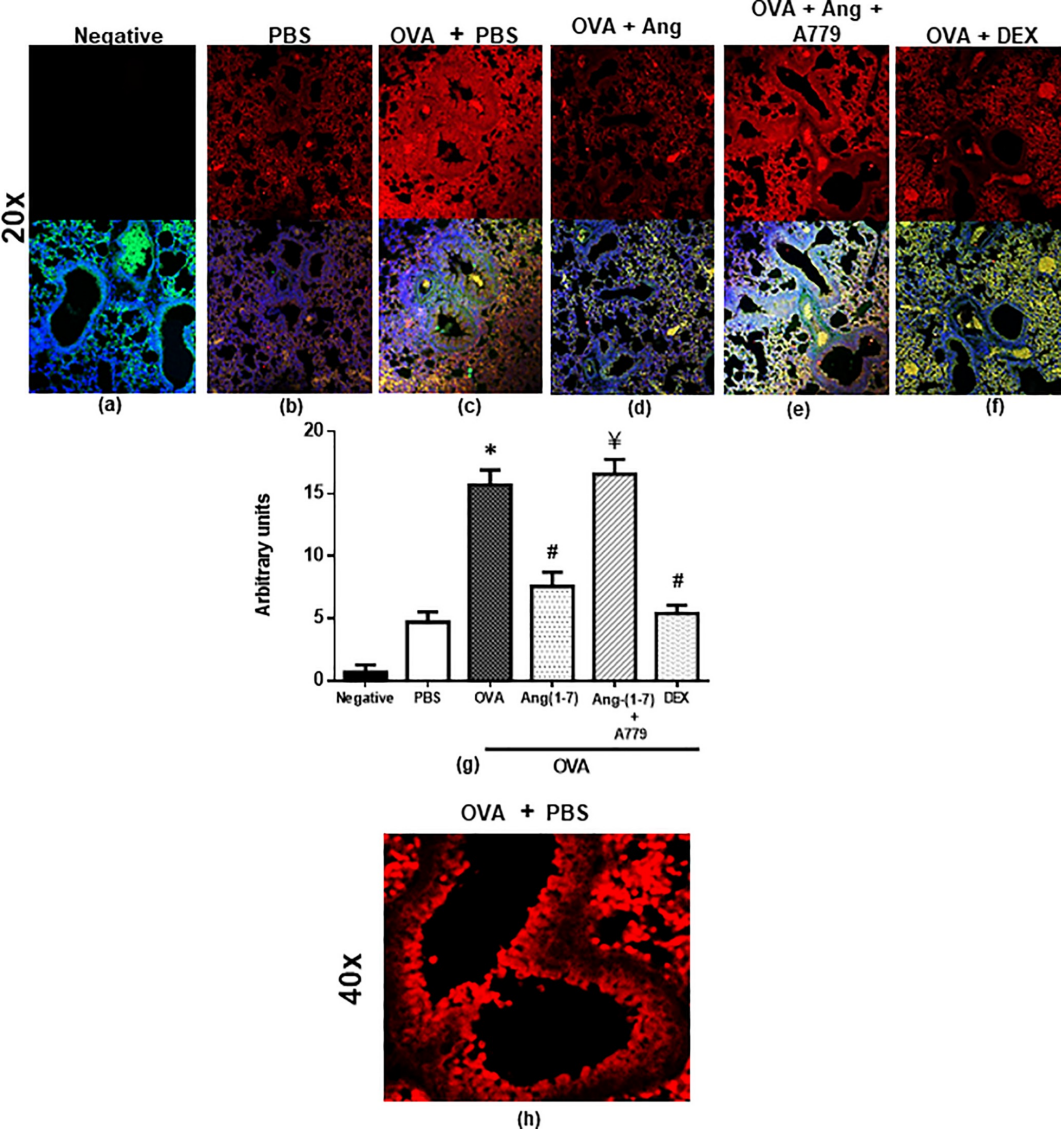
Immunofluorescent (Alexa Fluor) detection of phosphorylated Src shown on the upper panels are overlaid with DAPI stain on the lower panel to show lung tissue architecture. Lung sections were taken from different treatment groups and immunostained for phosphorylated Src (Fig 1(b–f)). Negative control (a); PBS/Veh (b); OVA/Veh (c); OVA/Ang-(1–7) (d); OVA/Ang-(1–7) + A779 (e) and OVA/Dex (f). PBS treated mice showed minimal pSrc (b). OVA challenge resulted in a significant increase in pSrc and this was inhibited following treatment with Ang-(1–7) (0.3 mg/kg) (c, d and g) and was comparable to the dexamethasone treated animals (f and g). Treatment with A779 inhibited the Ang-(1–7) (0.3 mg/kg)–induced decrease in pSrc (e and g). Quantitative assessment of fluorescence intensity of p-Src (Fig 1(g)) (arbitrary units). Data are expressed as mean ± SEM (*n* = 4–8). *P < 0.05 versus time-matched PBS-challenged mice. ^#^*P* < 0.05 versus time-matched ovalbumin-challenged mice. ^¥^ *P* < 0.05 versus time-matched OVA/Ang-(1–7) treated animals. Panel (h) is a higher magnification (x40) (x40) of the OVA challenged group to better show the localization of the stain.

OVA challenge resulted in a significant increase of approximately 3.3-fold in the phosphorylation of Src compared to PBS control as detected by immunofluorescence (P<0.05; Fig 1B, 1C and 1G). Treatment with Ang-(1–7) significantly inhibited the OVA-induced increase in Src phosphorylation by approximately 52.0% (P<0.05) and was somewhat comparable to the inhibition noted in the dexamethasone treatment group (P<0.05; Fig 1C, 1D, 1F and 1G). Treatment with A779, the MAS1 receptor antagonist, significantly reversed the effects of Ang-(1–7) on OVA-induced Src phosphorylation (P<0.05; Fig 1E and 1G). Fig 1H is a higher magnification (x40) immunofluorescence image of the OVA challenged mice lungs and shows that p-Src has a somewhat diffuse expression in both the airways and lung tissue.

#### Effect on EGFR

**Fig 2:**
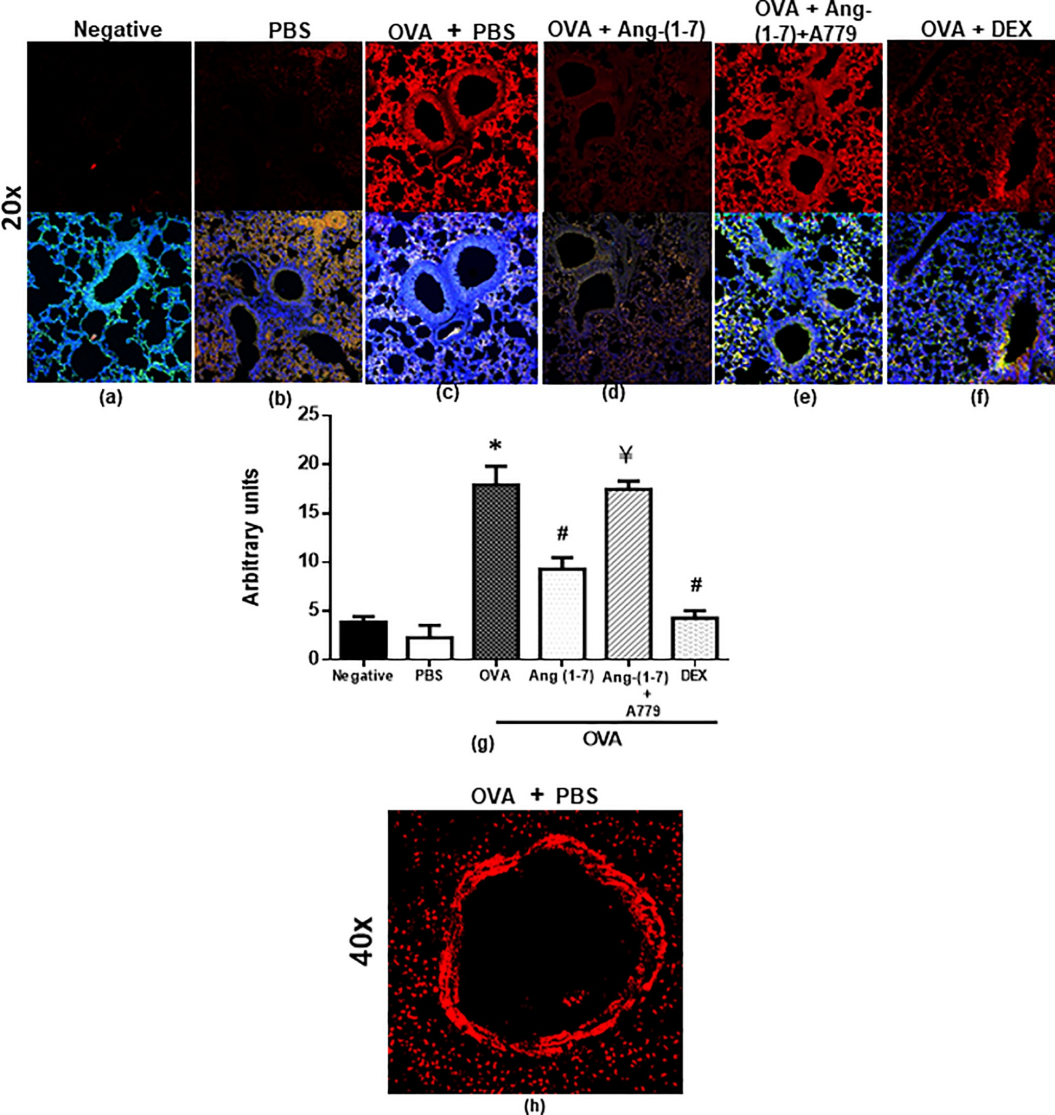
Immunofluorescent (Alexa Fluor) detection of phosphorylated EGFR shown on the upper panels are overlaid with DAPI stain on the lower panel to show lung tissue architecture. Lung sections were taken from different treatment groups and immunostained for pEGFR (Fig (1b–1f)). Negative control (a); PBS/Veh (b); OVA/Veh (c); OVA/Ang-(1–7) (d); OVA/Ang-(1–7) + A779 (e) and OVA/Dex (f). PBS treated mice showed minimal pEGFR (b). (a and b). OVA challenge resulted in a significant increase in pEGFR and this was inhibited following treatment with Ang-(1–7) (0.3 mg/kg) (c, d and g) and was comparable to the dexamethasone treated animals (f and g). Treatment with A779 inhibited the Ang-(1–7) (0.3 mg/kg)–induced decrease in pEGFR (e and g). Quantitative assessment of fluorescence intensity of pEGFR (Fig 1(g)) (arbitrary units). Data are expressed as mean ± SEM (*n* = 6–8). *P < 0.05 versus time-matched PBS-challenged mice. ^#^*P* < 0.05 versus time-matched ovalbumin-challenged mice. ^¥^ *P* < 0.05 versus time-matched OVA/Ang-(1–7) treated animals. Panel (h) is a higher magnification (x40) of the OVA challenged group to better show the localization of the stain.

OVA challenge resulted in a significant (P<0.05) 7.8-fold increase in the phosphorylation of EGFR compared to PBS control (Fig 2B, 2C and 2G). Treatment with Ang-(1–7) significantly (P<0.05) reduced the EGFR phosphorylation by about 48.0% (P<0.05; Fig 2C, 2D, 2F and 2G). The inhibitory effect of Ang-(1–7) on EGFR phosphorylation was completely reversed following treatment with A779 (P<0.05; Fig 2E and 2G). Fig 2H is a higher magnification (x40) immunofluorescence image of the OVA challenged mice and shows that whilst p-EGFR is expressed throughout the lung tissue and airways, there is a tendency for higher expression on the mucosal side of the airway.

#### Effect on ERK1/2

**Fig 3:**
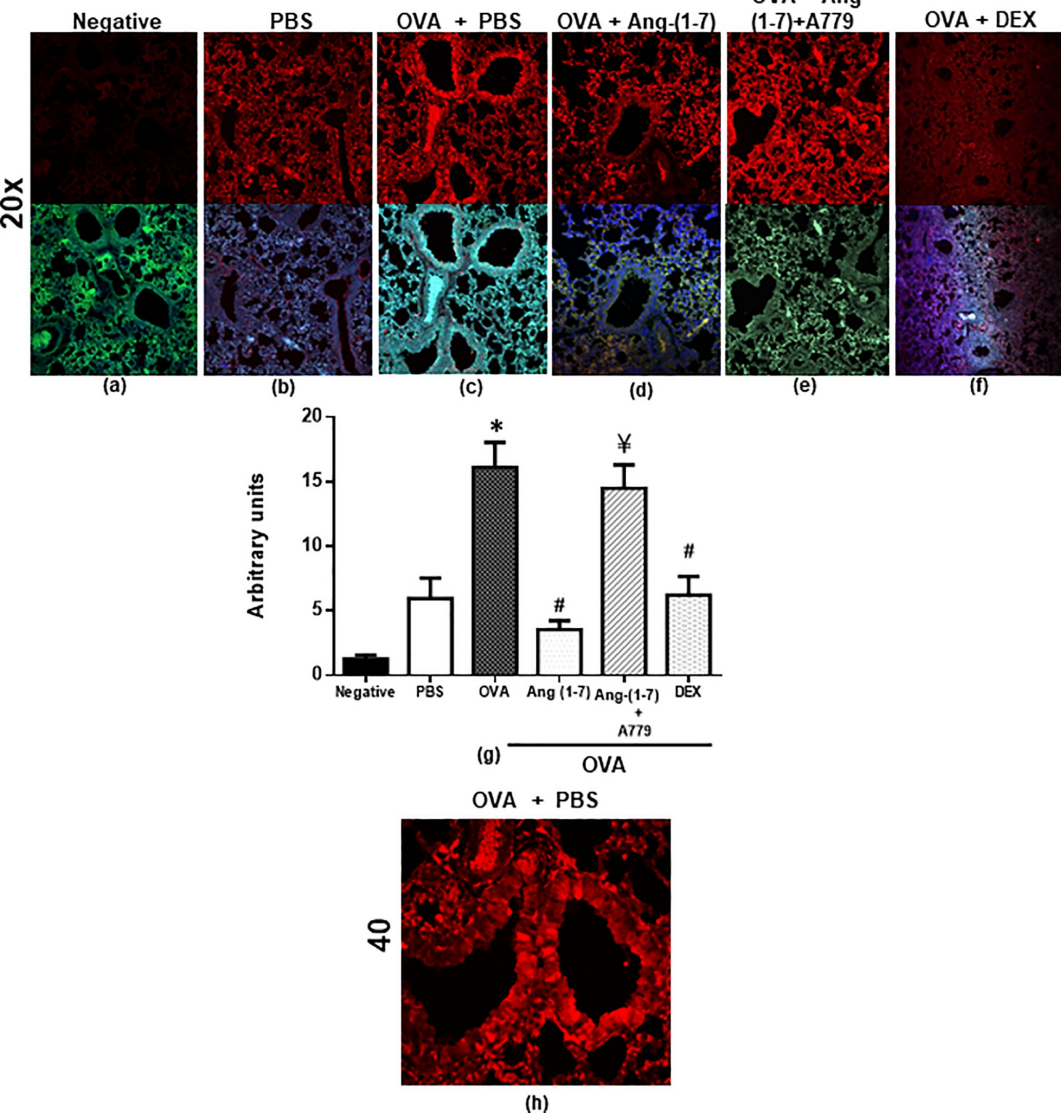
Immunofluorescent (Alexa Fluor) detection of phosphorylated ERK1/2 shown on the upper panels are overlaid with DAPI stain on the lower panel to show lung tissue architecture. Lung sections were taken from different treatment groups and immunostained for ERK1/2 (Fig (1b–1f)). Negative control (a); PBS/Veh (b); OVA/Veh (c); OVA/Ang-(1–7) (d); OVA/Ang-(1–7) + A779 (e) and OVA/Dex (f) (x 20 magnification). PBS treated mice showed minimal pERK1. OVA challenge resulted in a significant increase in ERK1/2 and this was inhibited following treatment with Ang-(1–7) (0.3 mg/kg) (c, d and g) and was comparable to the dexamethasone treated animals (f and g). Treatment with A779 inhibited the Ang-(1–7) (0.3 mg/kg)–induced decrease in ERK1/2 (e and g). Quantitative assessment of fluorescence intensity of ERK1/2 (Fig 1(g)) (arbitrary units). Data are expressed as mean ± SEM (*n* = 5–11). *P < 0.05 versus time-matched PBS-challenged mice. ^#^*P* < 0.05 versus time-matched ovalbumin-challenged mice. ^¥^ *P* < 0.05 versus time-matched OVA/Ang-(1–7) treated animals. Panel (h) is a higher magnification (x40) of the OVA challenged group to better show the localization of the stain.

OVA challenge resulted in a significant (P<0.05) 2.7-fold increase in the phosphorylation of ERK1/2 compared to PBS control (Fig 3B, 3C and 3G). Treatment with Ang-(1–7) also resulted in significant (P<0.05) 78.0% inhibition of ERK1/2 phosphorylation which was slightly greater than the inhibition (61%) noted following the dexamethasone treatment reduction (P<0.05; 1c, d, f and g). Again, treatment with A779 completely blocked the Ang-(1–7) mediated inhibition of the OVA-induced increase in ERK1/2 phosphorylation (P<0.05; Fig 3E and 3G). Fig 2H is a higher magnification (x40) immunofluorescence image of the OVA challenged mice and shows high expression of p-ERK throughout the airways.

### Effect of Ang-(1–7) on phosphorylation of Src, EGFR and ERK1/2

**Fig 4:**
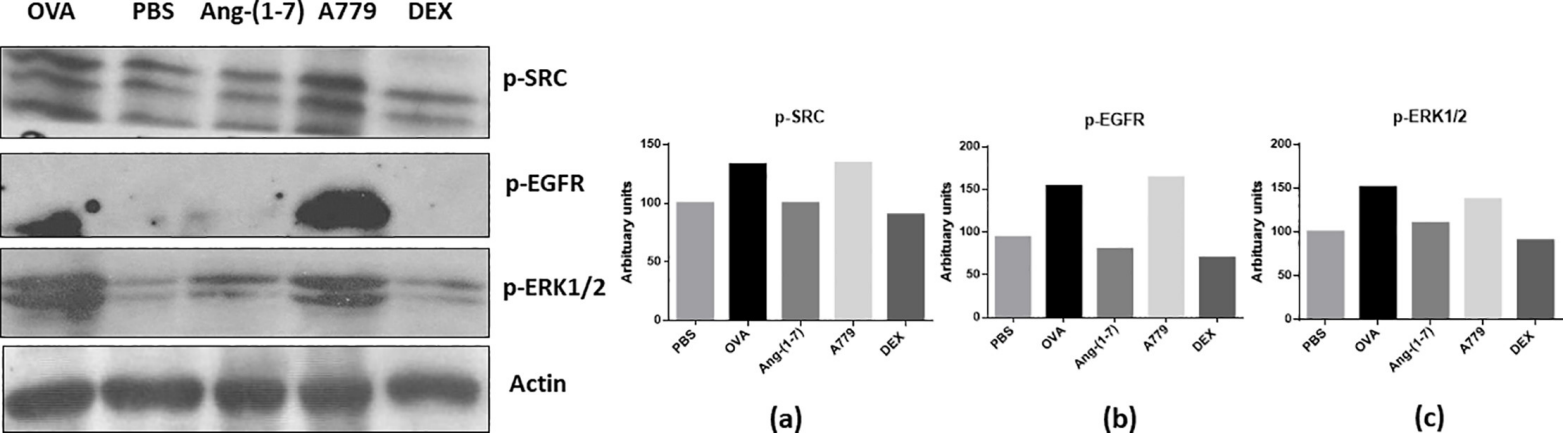
Western blot analysis of pSrc, pEGFR and p-ERK1/2 protein levels from lungs of PBS challenged mice pretreated with vehicle (PBS), ovalbumin challenged pretreated with vehicle (OVA), ovalbumin challenged pretreated with Ang-(1–7) (Ang-(1–7)), ovalbumin mice challenged pretreated with A779 + Ang-(1–7) (A779) and ovalbumin challenged pretreated with dexamethasone (DEX). The blots are representative of two similar but independent experiments (n = 3). Graphs a, b and c are densitometric quantification showing relative levels of p-Src, p-EGFR and p-ERK1/2, respectively (normalized to β-actin) of the shown blot.

Western blotting analysis of lung homogenate (Fig 4) confirmed the modulated levels of p-Src, p-EGFR and p-ERK1/2 seen in IF analysis (Figs 1, 2 and 3). OVA challenge resulted in a marked increase in p-Src, p-EGFR and p-ERK1/2 compared to PBS challenged mice (Fig 4). Treatment with Ang—(1–7) resulted in a clear inhibition of all of the phosphorylated proteins. In contrast, treatment with A779 blocked the Ang -(1–7) mediated inhibition whereas treatment with dexamethasone resulted in an inhibition of the OVA-induced increase in p-Src, p-EGFR and p-ERK1/2, similar to the effect of Ang-(1–7) (Fig 4).

### Effect of Ang-(1–7) on OVA- induced inflammatory cell influx

**Fig 5:**
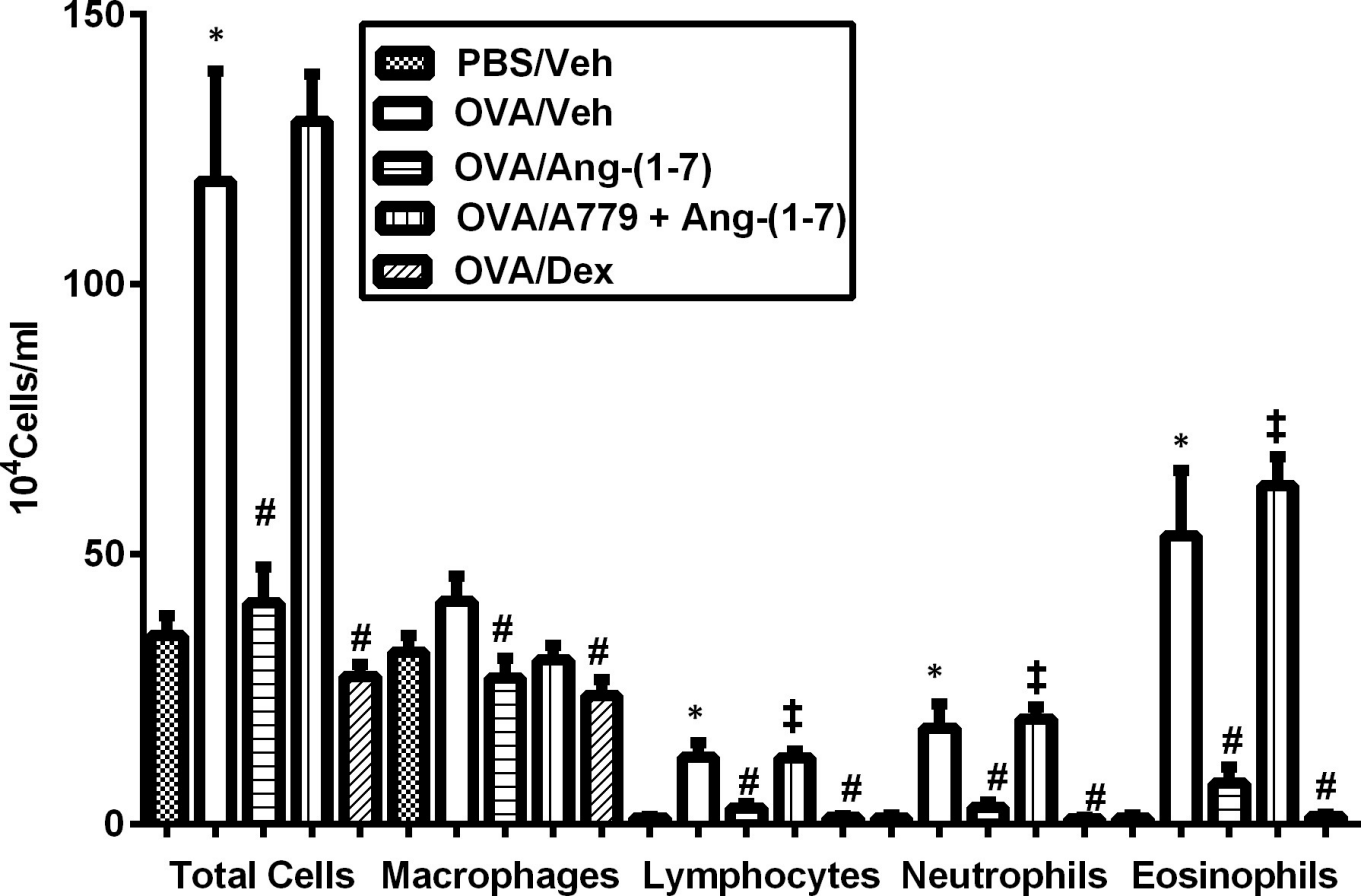
Effect of Ang-(1–7) (0.3 mg/kg; i.p) and A779 on ovalbumin-induced change in total BALF cell count, eosinophils, lymphocytes, neutrophils and macrophage. Treatment with A779 significantly attenuated the Ang-(1–7)-mediated inhibition of the ovalbumin-induced increase in total cell influx, eosinophils, lymphocytes and neutrophils in the airways. Data are expressed as mean ± SEM (*n* = 8–14). *P < 0.05 versus time-matched PBS-challenged mice. ^#^*P* < 0.05 versus time-matched ovalbumin-challenged mice.

OVA-sensitized and challenged animals had a significant increase in total cell count compared with the control group 24 h after the last challenge (119.1 ± 20.4 versus 35 ± 3.6 (×10^4^) cells/ml BAL fluid, respectively; P < 0.05; n = 14; Fig 5). Similarly, there were significant increases in the numbers of lymphocytes, neutrophils and eosinophils, but not macrophages (Fig 5). Treatment with Ang-(1–7) (0.3mg/kg; i.p.) significantly (P<0.05) decreased both total and the differential (lymphocytes, neutrophils and eosinophils) cell numbers to levels similar to those observed with dexamethasone treatment (1 mg/kg) (Fig 5). Furthermore, treatment with A779 (1 mg/kg; i.p.) significantly (P < 0.05) blocked the Ang-(1–7)-induced decrease in total cell count, eosinophils, lymphocytes and neutrophils compared with vehicle-treated mice (Fig 5).

### Effect of Ang-(1–7) on OVA—Induced histopathological changes

**Fig 6:**
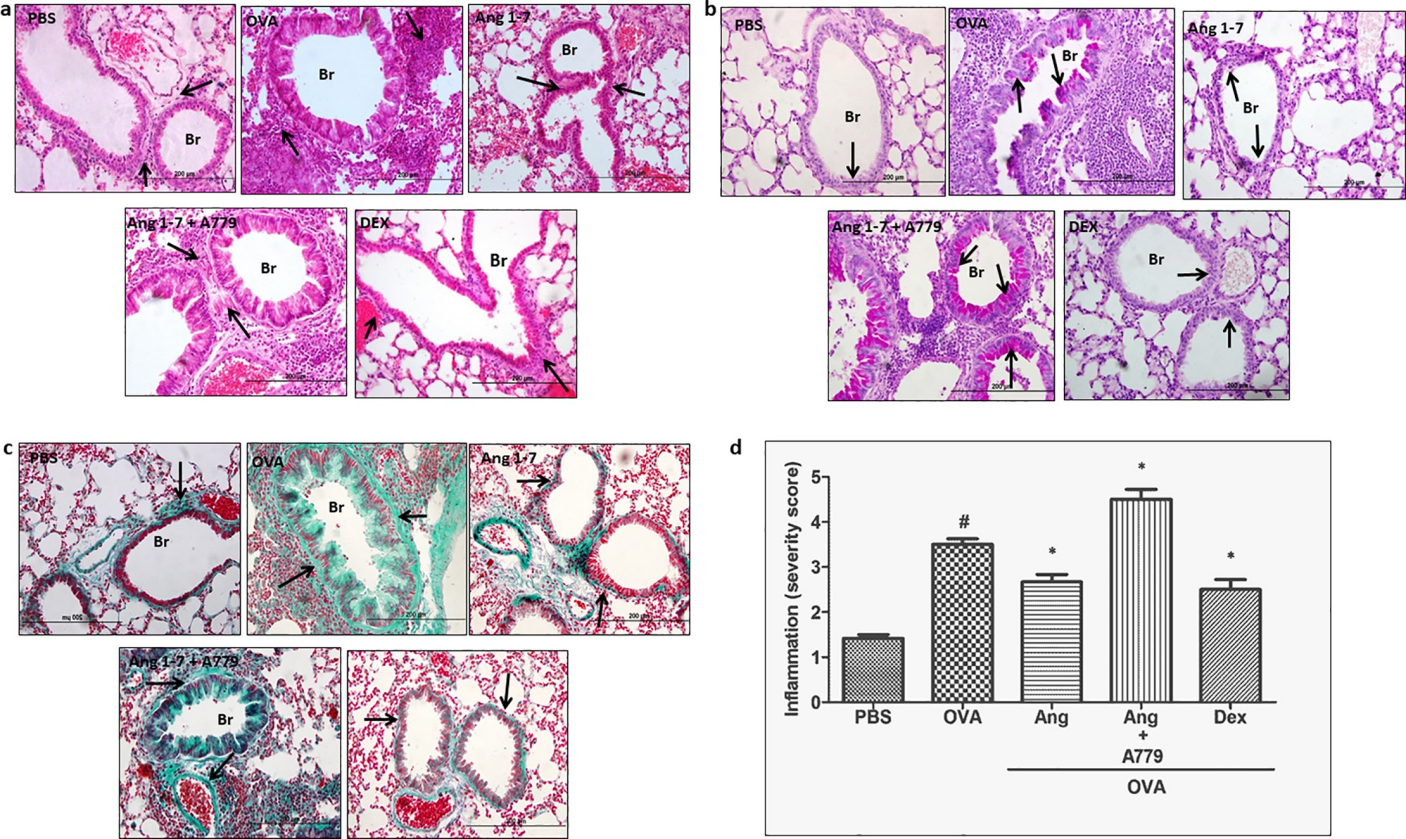
Representative low-magnification light photomicrographs display H&E staining (Fig 6(a)), Masson's Trichrome staining (Fig 6(b)) and PAS stain Fig 6(c)) of whole lung samples from PBS-challenged/ vehicle treated (*n* = 6) (PBS), OVA-challenged/ vehicle treated (*n* = 6) (OVA), OVA-challenged/Ang-(1–7) treated (0.3 mg/kg; *n* = 6) (Ang-1–7), OVA-challenged/Ang-(1–7) and A779 treated (0.3 mg/kg; *n* = 6 and 1mg/kg; *n* = 5; respectively) (Ang-1–7 + A779), OVA-challenged/dexamethasone treated (1 mg/kg; *n* = 6) (DEX). OVA-challenged/vehicle treated mice showed marked and significant peribronchial and perivascular inflammatory cell infiltrations (a) peribronchial and perivascular fibrosis (b) and bronchial mucus production and goblet cell hyper/metaplasia (c) compared with PBS-challenged vehicle treated mice. Treatment with Ang-(1–7) resulted in a significant reduction in the peribronchial and perivascular dark-staining inflammatory cell infiltration (a), peribronchial and perivascular fibrosis (b) and bronchial mucus production and goblet cell hyper/metaplasia (c) compared to the OVA – challenged mice and was comparable to PBS-challenged and OVA-challenged/dexamethasone treated mice. Effect of Ang-(1–7) (0.3 mg/kg) on inflammation severity score is shown in Fig 6(d). Data are expressed as mean ± SEM (*n* = 5–6). *P < 0.05 versus time-matched PBS-challenged mice. ^#^*P* < 0.05 versus time-matched ovalbumin-challenged mice. ‡*P* < 0.05 versus time-matched Ang-(1–7)-treated ovalbumin-challenged mice.

Airway remodeling was observed following OVA challenge in the OVA group as evidenced by severe perivascular and peribronchial inflammatory cell infiltration (H&E stain), peribronchial fibrosis (Masson’s Trichrome stain) and bronchial mucus production and goblet cell hyper/metaplasia (PAS stain) compared to the PBS challenged control group which had a normal airway morphology (Fig 6a 6b, 6c and 6d, P<0.05). Treatment with Ang-(1–7) (0.3mg/kg; i.p.) resulted in amelioration of OVA-induced inflammation with a significant reduction in the airway cellular influx, airway fibrosis, goblet cell hyper/metaplasia and mucus production, achieving almost normal histological appearance and indeed was as effective as dexamethasone treatment (1 mg/kg) (Fig 6a, 6b, 6c and 6d, P<0.05). These effects of Ang-(1–7) were completely reversed upon treatment with A779 (Fig 6a 6b, 6c and 6d, P<0.05).

### Effect of Ang-(1–7) on OVA—Induced airway hyperresponsiveness (AHR)

**Fig 7:**
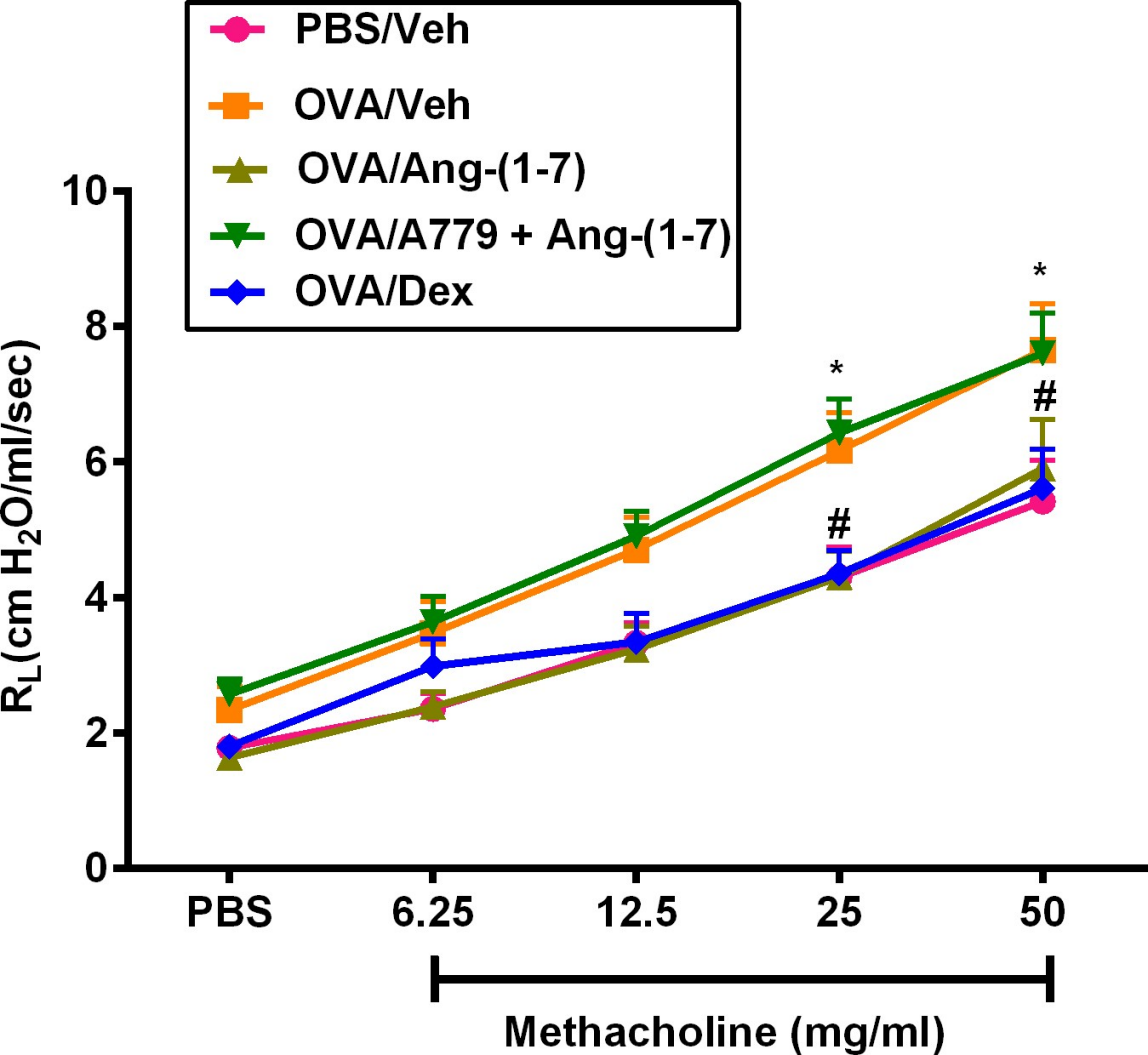
Effect of Ang-(1–7) (0.3 mg/kg), Ang-(1–7) (0.3 mg/kg) and A779 (1 mg/kg), and dexamethasone (1 mg/kg) on ovalbumin-induced AHR to inhaled methacholine. Lung function measurements were done 24 h after the last challenge. OVA challenged mice had significant AHR compared with the PBS/Veh group. Treatment with both Ang-(1–7) (0.3 mg/kg) and dexamethasone (1 mg/kg) both significantly reduced the OVA-induced AHR. Data are expressed as mean ± SEM (*n* = 12–19).

In these experiments we evaluated the effect of Ang-(1–7) treatment on the OVA-induced AHR. Twenty-four hours after the last intranasal OVA challenge of sensitized mice, there was a significant increase in airway responsiveness, characterized by an increase in lung resistance (R_L_) to methacholine in the OVA challenged mice compared to the PBS treated control mice and was significant at doses 25 and 50 mg/ml of methacholine compared (6.1 ± 0.6 and 7.6 ± 0.7 vs 4.3 ± 0.4 and 5.4 ± 0.6; P < 0.05; Fig 7). Treatment with Ang-(1–7) (0.3mg/kg) significantly reduced the average R_L_ in comparison with the OVA-challenged/vehicle-treated group at both the 25 and 50 mg/ml dose of methacholine (4.3 ± 0.4 and 5.9 ± 0.7 vs 6.1 ± 0.6 and 7.6 ± 0.7; cm H_2_O/ml per second P < 0.05; Fig 7), and was comparable to that of dexamethasone (1 mg/kg) treated group (4.4 ± 0.3 and 5.6 ± 0.6 cm H_2_O/ml per second) as it produced a significant reduction (P < 0.05) of AHR. On the other hand, treatment with A779 (1 mg/kg; i.p.) completely blocked the Ang-(1–7) induced reduction of the AHR

### Effect of Ang 1–7 treatment on neutrophil and eosinophil chemotaxis *ex vivo*

**Fig 8:**
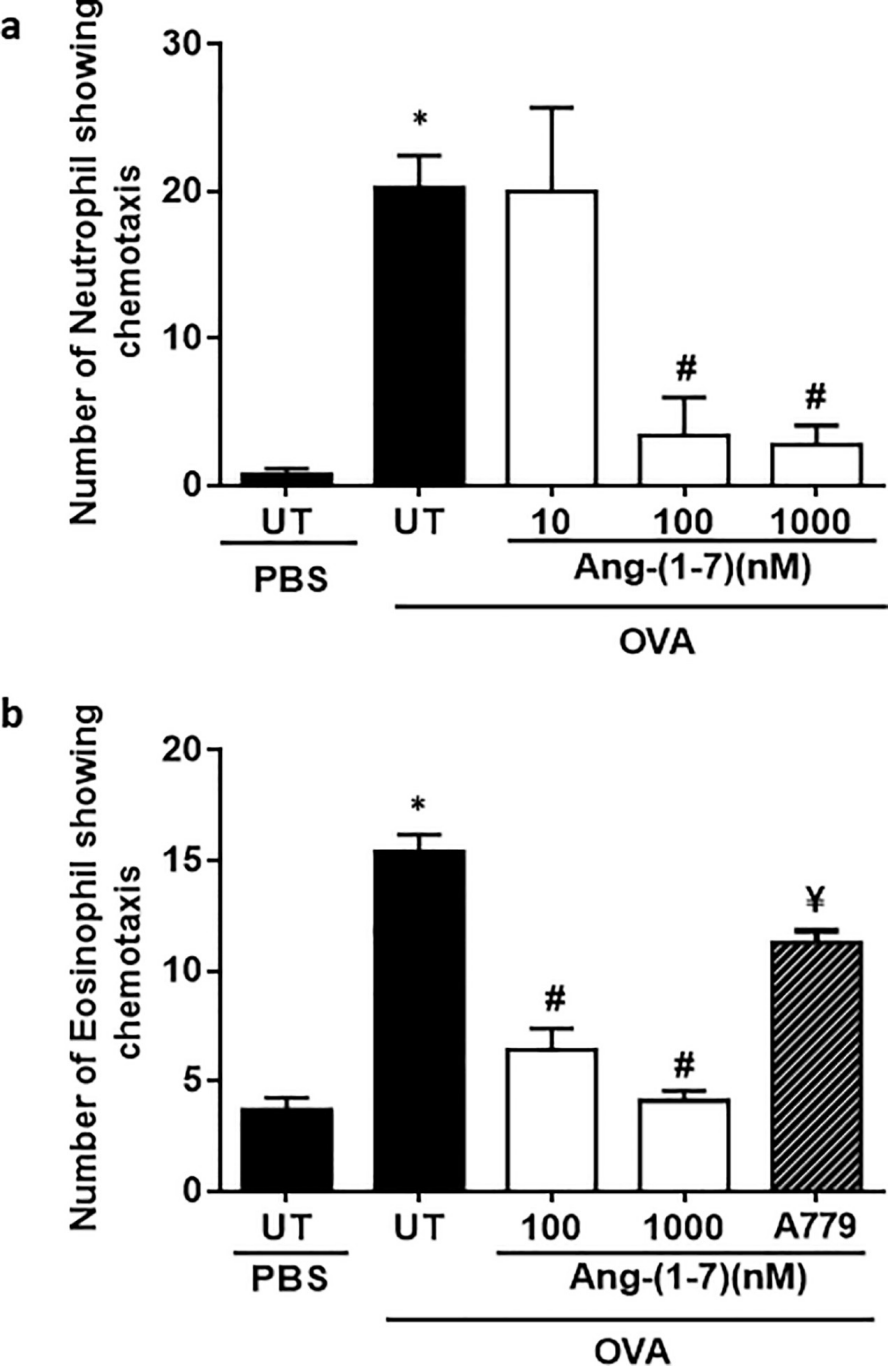
Effect of Ang (1–7) treatment on (a) neutrophil and (b) eosinophil chemotaxis towards BALF taken from either vehicle- or OVA-treated mice. Treatment with Ang-(1–7) inhibited both neutrophil and eosinophil migration. Treatment with A779 inhibited the eosinophil migration. Data are expressed as mean ± SEM (*n* = 5–8). *P < 0.05 *versus* time-matched PBS-challenged mice. ^#^*P*<0.05 *versus* time-matched OVA-challenged mice.

There was a significant increase in neutrophil chemotaxis towards BALF from OVA-treated mice compared to BALF taken from PBS challenged mice (UT) (P<0.05; Fig 8A). Ang 1–7 pretreatment (100–1000 nM) significantly reduced the OVA/BALF-induced neutrophil chemotaxis *ex vivo* (P<0.05; Fig 8A). Similarly, there was a significant increase in eosinophil chemotaxis towards BALF from OVA-treated mice compared to BALF from PBS challenged mice (UT) (P<0.05; Fig 8B). Again, pretreatment with Ang-(1–7) (100–1000 nM) dose dependently inhibited the OVA/BALF-induced eosinophil chemotaxis and this was significantly (P<0.05) inhibited following pretreatment with A779.

## Discussion

The major finding of this study is that the anti-inflammatory effects of Ang-(1–7) in an allergic murine model of asthma are mediated, at least in part, via the inhibition of Src and EGFR phosphorylation and consequent suppression of their downstream signaling effectors such as ERK1/2. Furthermore, we also showed that Ang-(1–7) directly inhibits neutrophil and eosinophil chemotaxis *ex vivo*. These results imply that the anti-inflammatory and anti-AHR effects of the Ang-(1–7)/MAS1 receptor axis are mediated, at least in part, via suppression of the Src/EGFR/ERK1/2 dependent signaling pathway.

The RAAS system has long been known to be a central regulator of heart and kidney homeostasis. Its activation has also been implicated in several disease states such as asthma, cardiovascular, renal and cancer and induces many pathophysiological effects such as vasoconstriction, cell proliferation, inflammation and fibrosis [49–51]. These effects are thought to be mediated mainly via the ACE/AngII/AT_1_R-axis. The recent discovery of new components of the RAAS such as angiotensin converting enzyme 2 (ACE2), Ang-(1–7) MAS1 receptor, Ang-(1–9) and alamandine has shed a different light on the biology of the RAAS system [52, 53]. In particular, the identification of ACEII/Ang -(1–7)/ MAS1 receptor axis reveals an interesting level of complexity whereby the RAAS system regulates itself. Indeed, there is now overwhelming evidence that ACEII/(Ang-1-7)/ MAS1 receptor axis acts as a counter-regulatory axis to the ACE/AngII/AT_1_R-axis receptor axis [54]. This is clearly exemplified in the cardiovascular system, particularly in blood pressure regulation and in cardiac pathologies [55–57]. This paradigm is also seen in several biological systems regulated by the RAAS system [54, 56], but the molecular mechanisms by which this is achieved remains unknown.

A major finding of our study is that treatment with Ang-(1–7) resulted in MAS1 receptor mediated inhibition of the OVA- induced EGFR phosphorylation. The Ang-(1–7) effects on EGFR were also associated with a significant reduction in the OVA-induced effects on the total and differential airway cellular influx, particularly eosinophil and neutrophils, which have roles in stable and severe asthma, respectively [2, 58]. There was also a decrease in OVA-induced perivascular and peribronchial inflammation, fibrosis and goblet cell hyper/metaplasia. These effects on the allergic phenotype are in agreement with recent findings from our laboratory, and that of others, which show that activation of the ACEII/(Ang-1-7)/ MAS1 receptor via administration of Ang-(1–7) or AVE 0991 (AVE), the non-peptide mimetic of the angiotensin (Ang)-(1–7), significantly reduced airway inflammation and remodeling [19, 20]. The anti-inflammatory action of the (Ang-1-7)/ MAS1 receptor was also demonstrated in a recent study with MAS1 knockout mice [21]. The inhibition of EGFR activation by Ang-(1–7) reported in this study is in agreement with our previous data showing that pretreatment with Ang-(1–7) prevents hyperglycemia-induced EGFR transactivation-dependent vascular complications [43]. A growing body of evidence shows that EGFR-dependent signaling plays a significant role in asthma [59–61]. We and others have also recently reported in animal models, and in clinical studies, that increased EGFR activity is associated with the asthma phenotype. Our current data also shows that unlike p-Src and p-ERK1/2, there is a clear trend for an increase in p-EGFR in the mucosal side of the airways, which is in-line with findings demonstrating increased EGFR immunoreactivity in the bronchial epithelium [25]. The important role that EGFR plays in asthma is also underscored by studies showing that tyrosine kinase inhibitors such as gefitnib, erlotinibin and AG1478, inhibit both EGFR phosphorylation and asthma features such as cellular influx and airway remodeling and AHR [4, 27, 62].

The importance of EGFR signaling has also been noted in chronic obstructive pulmonary disease (COPD) where EGFR activity is increased and is associated with mucosal cell metaplasia—an important pathophysiological feature in both asthma and COPD [63]. Inhibition of EGFR activation is likely to be of clinical importance in the treatment of chronic airway inflammatory diseases [64]. However, one of the issues with EGFR inhibitors is their extensive side effect profile [65] which may be drug and/or receptor specific, as well as a potential for drug resistance [66], as is typical in cancer patients receiving EGFR inhibitors. The development of stable long acting MAS1 agonists may be a more effective alternative to EGFR inhibitors as potential anti-inflammatory therapy for asthma and other diseases.

Our data also show that treatment with Ang-(1–7) significantly reduced the OVA-induced increased phosphorylation of Src kinase, which appeared to be diffusely expressed in the lung tissue and the airways. This activation was fully reversed following treatment with the MAS1 receptor blocker A779. These data clearly suggest that activation of the Ang-(1–7)/MAS1 receptor axis has an inhibitory effect on Src kinase. This finding is in line with our recent study showing that, in hyperglycemic diabetic animal model, Ang-(1–7) inhibits enhanced Src phosphorylation and the diabetes-associated changes in the mesenteric vasculature [43, 67]. The importance of Src in asthma was also highlighted in our recent study where we showed that its inhibition results in the inhibition of airway inflammation, airway remodeling and AHR [4]. Importantly, inhibition of Src was associated with significant inhibition of EGFR activation suggesting that Src kinase is indeed upstream of EGFR. The exact role of the Src kinase family in asthma is still not fully understood. Nonetheless, Src kinases have been shown to play a role in the signaling pathways for critically important receptors in asthma such the T cell receptor (TCR) and the high affinity receptor for IgE (FcεRI) phenotype [68, 69] and thus, may act as an important signaling hub in the pathogenesis of this respiratory condition. Further, the inhibition of OVA-induced phosphorylation of both EGFR and Src kinase by Ang-(1–7) implies that this may be part of a broad-based anti-inflammatory effect possibly via inhibition of several pro-inflammatory pathways. However, that SU 6656, a selective Src family of kinase inhibitors, also prevented EGFR activation [4] suggests that a likely scenario is that Ang-(1–7) inhibits Src activation which in turn inhibits EGFR and its downstream signaling effectors such as ERK1/2.

We showed that treatment with Ang-(1–7) significantly inhibited the phosphorylation of ERK1/2, an effect that was reversed by treatment with the MAS1 receptor blocker A779. It is of interest to note that the effects of Ang-(1–7) on both EGFR and ERK1/2 were comparable to the dexamethasone treatment. ERK1/2 dependent signaling pathway has been shown to mediate important pathophysiological effects in models of asthma [19, 70, 71]. Indeed, we have recently shown that ERK1/2, in addition to PI3K/AKT, is an important signaling effector molecule downstream of EGFR and its selective blockade results in reduction of the asthma phenotype [4]. Interestingly, inhibition of ERK1/2 was more effective than inhibition of either Src or EGFR, implying that downstream inhibition is more effective than upstream pathway. Taken together, these data support the notion that the beneficial effects of Ang-(1–7) in a murine model of asthma involve, at least in part, an inhibition of the deleterious effects of increased activity of EGFR and its downstream signaling pathways.

Our data also showed that Ang-(1–7) can have direct effects on cell chemotaxis. For example, *ex vivo* neutrophil chemotaxis towards BALF from OVA-challenged mice was significantly inhibited by exogenous Ang-(1–7) application. This is in agreement with our previous study showing that Ang-(1–7) inhibited WKYMVm peptide induced neutrophil influx [46]. Moreover, a recent study has shown that pretreatment with Ang-(1–7) significantly reduces leukocyte adhesion and extravasation in diabetic mice *in vivo*, and also prevented the hyperglycemia-induced increase in adhesion molecules (ICAM-1 and VCAM-1) as well as neutrophil adhesion *in vitro* [72]. Similar to the effects on neutrophils, Ang-(1–7) also significantly inhibited the *ex vivo* eosinophil chemotaxis to BALF from our OVA challenged mice and this was reversed following treatment with A779. It is of interest to note that Ang-(1–7) has also been reported to promote eosinophilic resolution at least partly via induction of eosinophil apoptosis indicating the presence of the MAS1 receptor on eosinophils which when activated can directly modulate cellular functions such as chemotaxis and apoptosis [73]. Together, the data show that the inhibitory actions of Ang-(1–7) on cell migration can be mediated directly (Fig 8).

Our data also show that Ang-(1–7) suppresses the allergen-induced AHR. Increased airway responsiveness is an important clinical feature of asthma that is not easily amenable to asthma therapy [74]. Several studies have suggested that both airway inflammation and airway sensory hyper-excitability may underlie AHR, [75, 76]. Activation of the ACEII/(Ang-1-7)/ MAS1 pathway prevents the development of AHR but it is not clear whether this effect is due to inhibition of Src/EGFR/ ERK1/2 pathway or inhibition of another pro- inflammatory pathway.

In conclusion, the data presented in this study shows that inhibition of Src/EGRF/ERK1/2 is one of the mechanisms by which the Ang-(1–7)/ MAS1 receptor axis mediates its anti-inflammatory and anti-AHR effects in inflammatory diseases such as asthma.

## Supporting information

S1 Fig Immunoflorescence images for pSrc for the different groups. (PDF)

S2 Fig Immunoflorescence images for pEGFR for the different groups. (PDF)

S3 Fig Immunoflorescence images for pERK1/2 for the different groups. (PDF)

S4 Fig Western blot analysis of pSrc, pEGFR and pERK1/2 for the different groups. (PDF)

S1 Table Total cell numbers for the different groups. (PDF)

S2 Table Macrophage cell numbers for the different groups. (PDF)

S3 Table Lymphocyte cell numbers for the different groups. (PDF)

S4 Table Neutrophil cell numbers for the different groups. (PDF)

S5 Table Eosinophil cell numbers for the different groups. (PDF)

S6 Table Histological scores for the different groups. (PDF)

S7 Table RL values for the different groups—AHR experiment. (PDF)

S8 Table Eosinophil chemotaxis data for the different groups. (PDF)
