# Supplementary figures and images for: Ang-(1-7)/ MAS1 receptor axis inhibits allergic airway inflammation via blockade of Src-mediated EGFR transactivation in a murine model of asthma
Source: PLoS One. 2019 Nov 1;14(11):e0224163. doi: 10.1371/journal.pone.0224163 (PMC6824568; doi:10.1371/journal.pone.0224163)

**S1 Fig: Immunofluorescence images for pSrc for the different groups**

Negative:

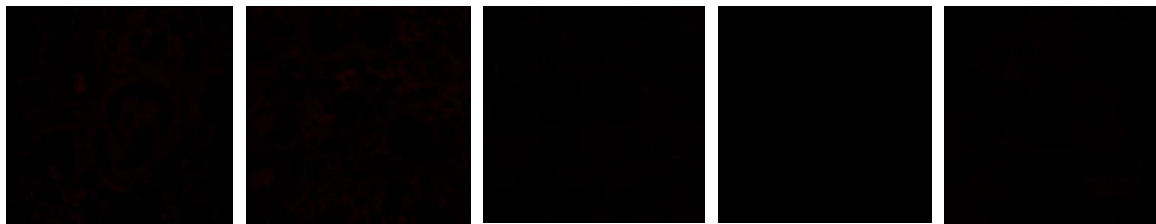

PBS:

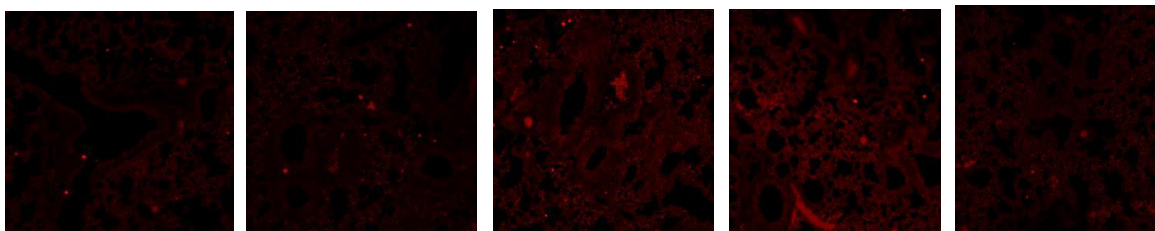

OVA

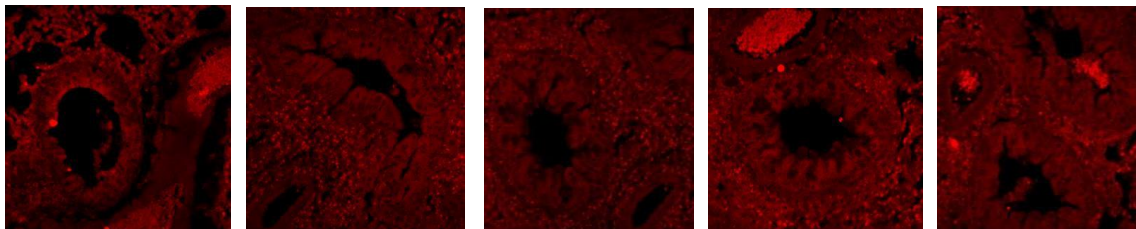

Ang(1-7)

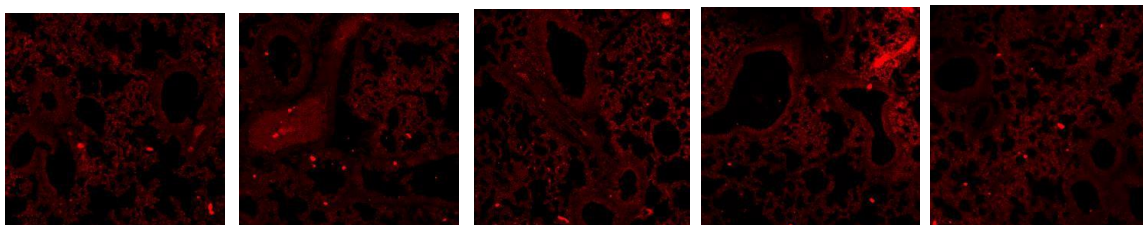

A779:

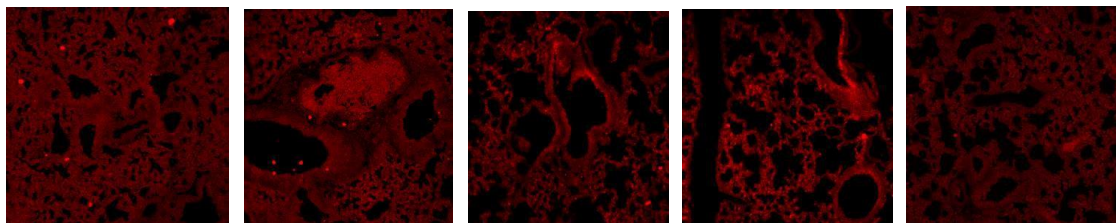

DEX:

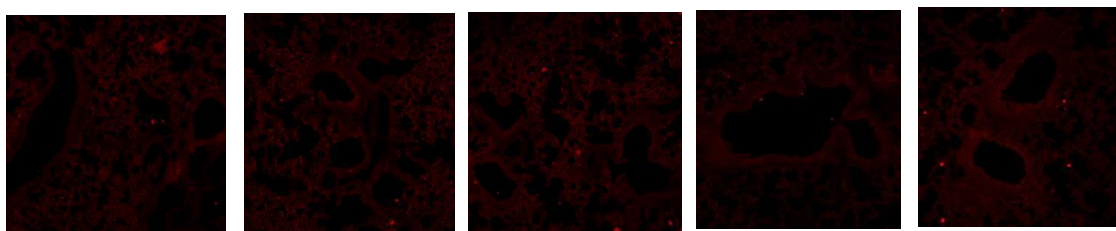

Supplement: S1 Fig — (PDF) [file pone.0224163.s001.pdf]

**S2 Fig: Immunoflorescence images for pEGFR for the different groups**

**Negative:**

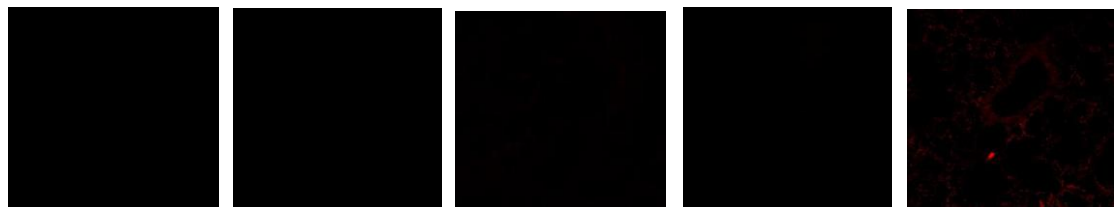

**PBS:**

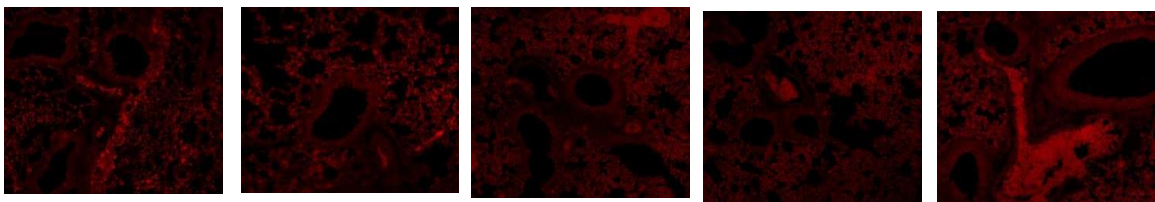

**OVA**

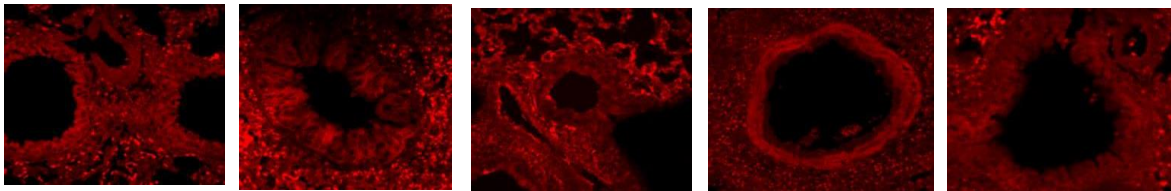

**Ang (1-7)**

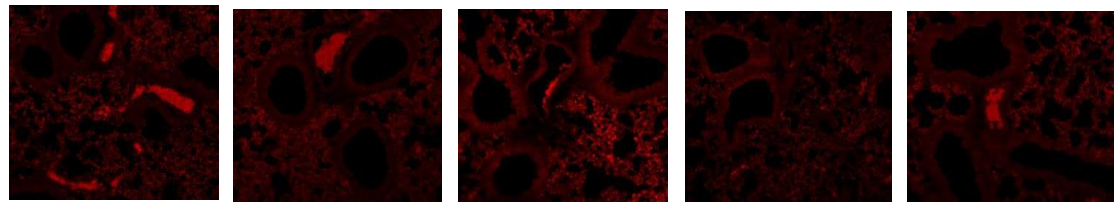

**A779:**

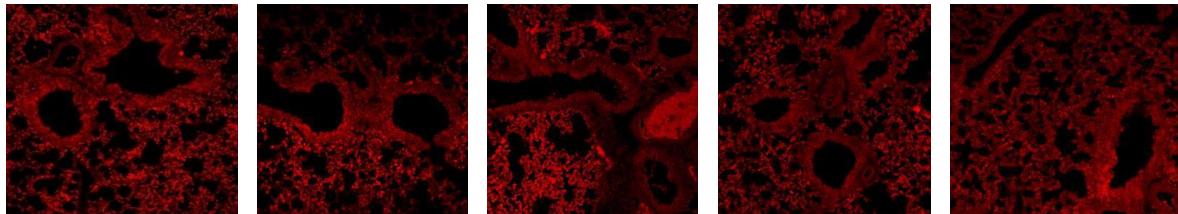

**DEX:**

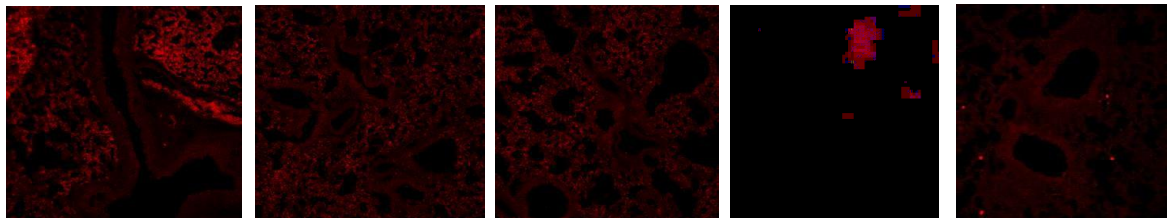

Supplement: S2 Fig — (PDF) [file pone.0224163.s002.pdf]

**S3 Fig: Immunofluorescence images for pERK1/2 for the different groups**

**Negative:**

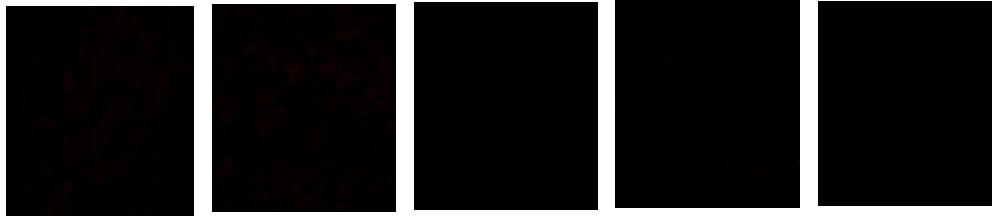

**PBS:**

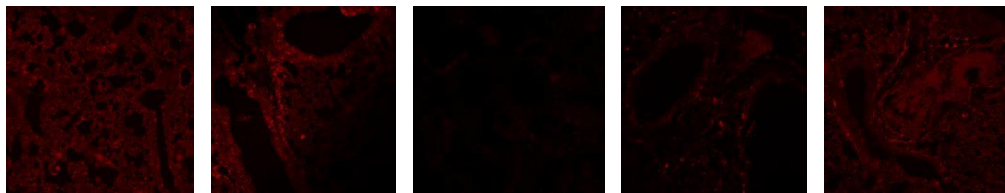

**OVA:**

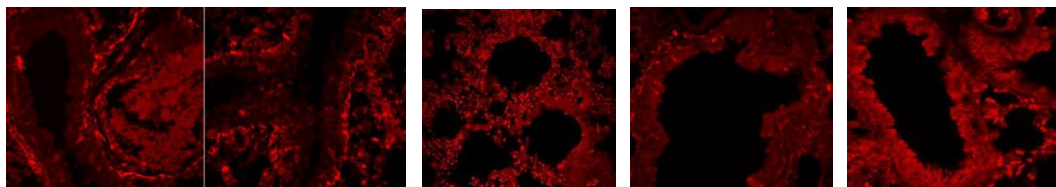

**Ang(1-7):**

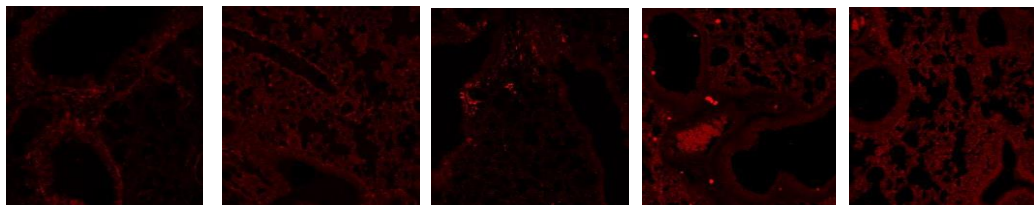

**A779:**

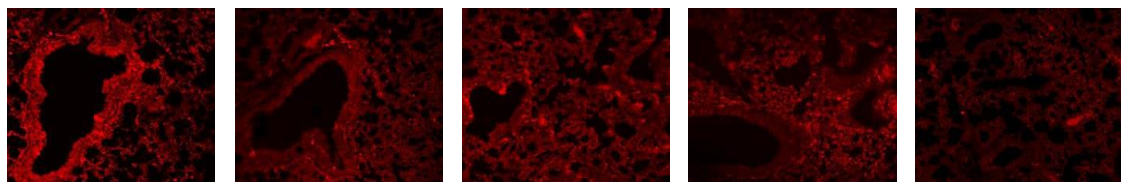

**DEX:**

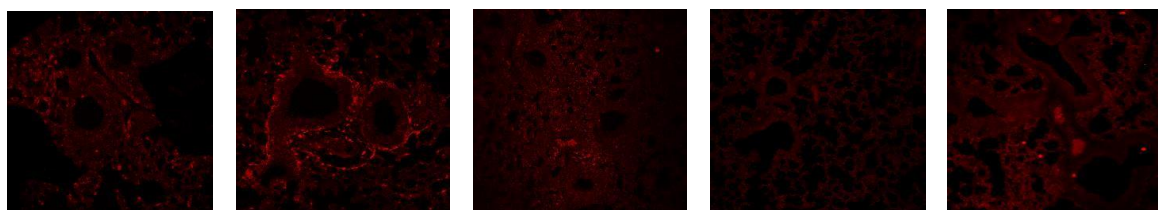

Supplement: S3 Fig — (PDF) [file pone.0224163.s003.pdf]

**S4 Fig. Western blot analysis of pSrc, pEGFR and pERK1/2 for the different groups**

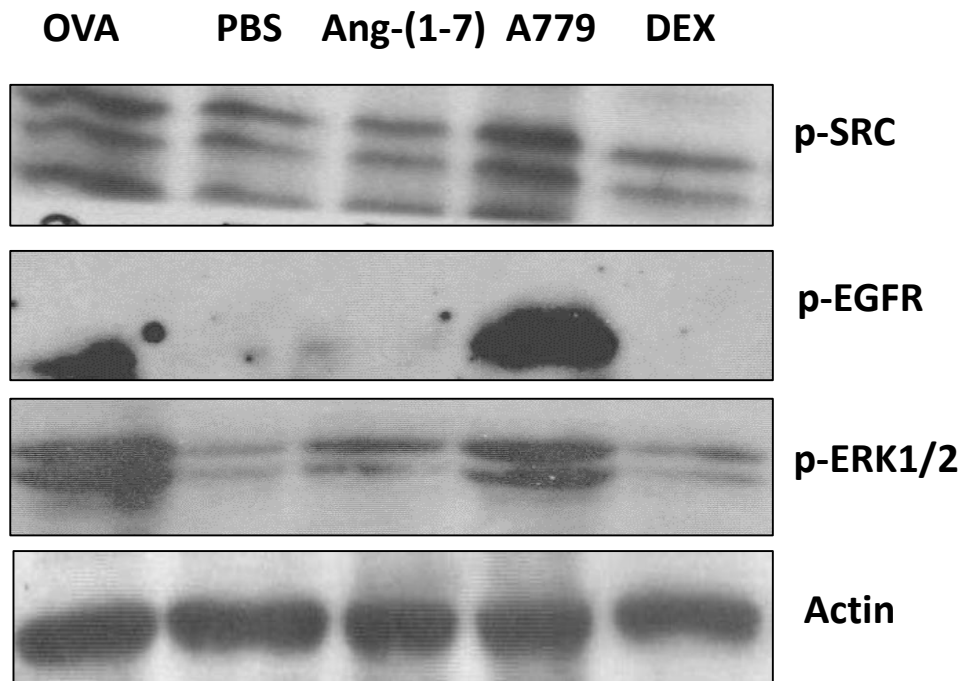

Supplement: S4 Fig — (PDF) [file pone.0224163.s004.pdf]
