# Supplementary material for: Ang-(1-7)/ MAS1 receptor axis inhibits allergic airway inflammation via blockade of Src-mediated EGFR transactivation in a murine model of asthma
Source: PLoS One. 2019 Nov 1;14(11):e0224163. doi: 10.1371/journal.pone.0224163 (PMC6824568; doi:10.1371/journal.pone.0224163)
Supplement: S1 Table — (PDF) [file pone.0224163.s005.pdf]

S1 Table: Total cell numbers for the different groups

Sample number	PBS	OVA	Ang(1-7)	A779 + Ang(1-7)	Dex
**1**	28.275000	102.950000	18.925000	130.350000	28.010000
**2**	45.285000	327.350000	48.130000	150.350000	29.180000
**3**	54.410000	14.455000	50.890000	152.900000	38.525000
**4**	20.340000	104.900000	22.245000	62.870000	28.975000
**5**	27.885000	94.135000	31.890000	115.550000	26.450000
**6**	29.770000	32.630000	67.510000	93.595000	24.785000
**7**	43.235000	169.400000	63.155000	114.150000	17.290000
**8**	46.460000	76.285000	25.700000	145.550000	34.695000
**9**	31.065000	116.350000		164.950000	18.710000
**10**	23.175000	116.500000		120.700000	
**11**		76.725000		152.300000	
**12**		95.580000		159.400000	
**13**		188.200000			
**14**		152.350000			
**MEAN**	**34.990000**	**119.129300**	**41.055600**	**130.222100**	**27.402220**
**SEM**	**3.613412**	**20.365900**	**6.679400**	**8.765100**	**2.262228**
