# Supplementary material for: Ang-(1-7)/ MAS1 receptor axis inhibits allergic airway inflammation via blockade of Src-mediated EGFR transactivation in a murine model of asthma
Source: PLoS One. 2019 Nov 1;14(11):e0224163. doi: 10.1371/journal.pone.0224163 (PMC6824568; doi:10.1371/journal.pone.0224163)
Supplement: S2 Table — (PDF) [file pone.0224163.s006.pdf]

S2 Table: Macrophage cell numbers for the different groups

Sample number	PBS	OVA	Ang(1-7)	A779 + Ang(1-7)	Dex
**1**	26.43712	33.4587	11.6388	22.1595	22.4080
**2**	37.81298	65.4700	40.9105	33.8287	33.5167
**3**	42.16775	24.1270	38.6764	17.2892	26.6570
**4**	19.3230	45.6554	15.0153	30.0430	15.9932
**5**	25.79362	15.6624	27.5848	28.0785	29.4907
**6**	41.07325	55.0550	30.7170	23.4007	14.9680
**7**	43.2078	62.1722	30.6301	47.3037	
**8**	29.8224	41.3042	21.7165	34.6395	
**9**	21.43688	37.8625		34.3995	
**10**		26.8537		33.5060	
**11**		30.5856			
**12**		57.4010			
**MEAN**	**31.897200**	**41.300670**	**27.111220**	**30.464850**	**23.838960**
**SEM**	**3.099081**	**4.635376**	**3.695047**	**2.657007**	**3.032273**
