# Supplementary material for: Ang-(1-7)/ MAS1 receptor axis inhibits allergic airway inflammation via blockade of Src-mediated EGFR transactivation in a murine model of asthma
Source: PLoS One. 2019 Nov 1;14(11):e0224163. doi: 10.1371/journal.pone.0224163 (PMC6824568; doi:10.1371/journal.pone.0224163)
Supplement: S3 Table — (PDF) [file pone.0224163.s007.pdf]

S3 Table: Lymphocyte cell numbers for the different groups

**Sample number**	**PBS**	**OVA**	**Ang(1-7)**	**A779 + Ang(1-7)**	**Dex**
**1**	0.989625	7.206500	1.419375	12.383250	0.560200
**2**	2.490675	36.008500	1.925200	18.042000	2.696750
**3**	2.448450	9.441000	4.071200	8.487450	0.434625
**4**	0.711900	4.706750	1.890825	12.132750	0.950950
**5**	0.975975	3.752450	0.956700	9.359500	2.255175
**6**	0.864700	15.246000	5.063250	9.702750	0.841950
**7**	1.626100	4.958525	7.578600	8.733000	
**8**	0.465975	8.144500	1.542000	14.845500	
**9**	0.811125	16.310000		9.656000	
**10**		8.439750		19.799000	
**11**		9.558000			
**12**		25.407000			
**MEAN**	**1.264947**	**12.431580**	**3.055894**	**12.314120**	**1.289942**
**SEM**	**0.2500888**	**2.775514**	**0.8186196**	**1.270870**	**0.3868887**
