# Supplementary material for: Ang-(1-7)/ MAS1 receptor axis inhibits allergic airway inflammation via blockade of Src-mediated EGFR transactivation in a murine model of asthma
Source: PLoS One. 2019 Nov 1;14(11):e0224163. doi: 10.1371/journal.pone.0224163 (PMC6824568; doi:10.1371/journal.pone.0224163)
Supplement: S4 Table — (PDF) [file pone.0224163.s008.pdf]

S4 Table: Neutrophil cell numbers for the different groups

Sample number	PBS	OVA	Ang(1-7)	A779 + Ang(1-7)	Dex
**1**	0.424125	15.442500	2.838750	19.552500	2.660950
**2**	2.264250	60.559750	1.684550	21.049000	0.385250
**3**	4.624850	14.686000	2.544500	9.116150	1.303875
**4**	0.203400	5.177425	2.002050	19.643500	0.172900
**5**	0.836550	4.568200	0.956700	7.019625	0.693900
**6**	0.864700	16.093000	8.776300	27.396000	0.935500
**7**	1.161500	1.525700	5.999725	17.466000	
**8**	0.310650	20.361250	0.899500	32.990000	
**9**	0.463500	20.970000		18.105000	
**10**		9.207000		22.845000	
**11**		15.770700			
**12**		30.112000			
**MEAN**	**1.239281**	**17.872790**	**3.212759**	**19.518280**	**1.025396**
**SEM**	**0.4717419**	**4.515895**	**0.9778696**	**2.421208678**	**0.365472**
