# Supplementary material for: Ang-(1-7)/ MAS1 receptor axis inhibits allergic airway inflammation via blockade of Src-mediated EGFR transactivation in a murine model of asthma
Source: PLoS One. 2019 Nov 1;14(11):e0224163. doi: 10.1371/journal.pone.0224163 (PMC6824568; doi:10.1371/journal.pone.0224163)
Supplement: S5 Table — (PDF) [file pone.0224163.s009.pdf]

S5 Table: Eosinophil cell numbers for the different groups

**Sample number**	**PBS**	**OVA**	**Ang(1-7)**	**A779 + Ang(1-7)**	**Dex**
**1**	0.424125	46.842250	3.028000	76.254750	2.380850
**2**	2.717100	165.311800	3.609750	77.430250	1.926250
**3**	5.168950	56.646000	5.597900	27.977150	0.579500
**4**	0.101700	38.595350	3.336750	53.730750	0.172900
**5**	0.278850	8.646950	2.391750	49.137380	2.255175
**6**	0.432350	83.006000	22.953400	53.650500	1.964550
**7**	0.464600	7.628500	18.946500	72.047250	
**8**	0.465975	46.540000	1.542000	82.475000	
**9**	0.463500	41.357500		58.539500	
**10**		32.224500		76.150000	
**11**		39.665700			
**12**		75.280000			
**MEAN**	**1.168572**	**53.478710**	**7.675756**	**62.739250**	**1.546538**
**SEM**	**0.5641286**	**12.022430**	**2.949753**	**5.405030**	**0.3803157**
