# Supplementary material for: Ang-(1-7)/ MAS1 receptor axis inhibits allergic airway inflammation via blockade of Src-mediated EGFR transactivation in a murine model of asthma
Source: PLoS One. 2019 Nov 1;14(11):e0224163. doi: 10.1371/journal.pone.0224163 (PMC6824568; doi:10.1371/journal.pone.0224163)
Supplement: S6 Table — (PDF) [file pone.0224163.s010.pdf]

S6 Table: Histological scores for the different groups

Sample number	PBS	OVA	Ang(1-7)	A779 + Ang(1-7)	Dex
**1**	1.5	3.5	2.5	4.5	2.5
**2**	1.5	3	3	5	2.5
**3**	1.5	3.5	3	5	2.5
**4**	1	3.5	2	4	1.5
**5**	1.5	4	2.5	4	3
**6**	1.5	3.5	3		3
**MEAN**	**1.416667**	**3.5**	**2.666667**	**4.5**	**2.5**
**SEM**	**0.083333**	**0.129099445**	**0.166667**	**0.223607**	**0.223606798**
