# Supplementary material for: Ang-(1-7)/ MAS1 receptor axis inhibits allergic airway inflammation via blockade of Src-mediated EGFR transactivation in a murine model of asthma
Source: PLoS One. 2019 Nov 1;14(11):e0224163. doi: 10.1371/journal.pone.0224163 (PMC6824568; doi:10.1371/journal.pone.0224163)
Supplement: S7 Table — (PDF) [file pone.0224163.s011.pdf]

S7 Table: RL values for the different groups - AHR experiment

veh/PBS	PBS	6.25 mg/ml	12.5 mg/ml	25 mg/ml	50 mg/ml
1	1.337270667	1.540013776	2.599755613	2.903272575	4.883938969
2	1.431466209	2.418114295	2.523706132	4.107814183	4.498075959
3	2.160097974	2.592211224	3.801962884	5.127875137	5.94311168
4	1.436737224	1.566800753	3.258485077	4.15450659	4.791815456
5	1.66373463	1.855312317	3.518586917	4.235242367	6.391606871
6	1.449804249	2.009123141	3.558184773	3.25631772	4.652179219
7	1.676748343	1.6520531	4.65039686	6.982676945	6.295341867
8	0.527686348	0.736908749	0.731565357	0.801485252	0.993967767
9	1.18187113	1.232530737	2.093114858	3.394763745	4.877247876
10	1.924483579	2.265333842	3.129502519	3.607253443	4.861418753
11	2.174766332	3.062046903	3.981705071	4.59296753	6.135859835
12	1.376489375	3.485030576	5.971920945	7.397451235	11.97776841
13	1.93915418	3.617592835	4.258942213	7.878673824	9.947985714
14	1.550643808	1.853040245	1.612925663	2.618565736	3.226290083
15	3.54223698	4.294963849	4.557152777	5.29361187	4.463266614
16	1.657945557	2.143085659	2.561350683	2.663769948	3.788096689
17	3.188937685	3.642174298	3.730930522	4.005679382	4.331939891
**Average**	**1.777651428**	**2.350960959**	**3.325893463**	**4.295407499**	**5.415288921**
**STDEV**	**0.714226131**	**0.974142543**	**1.250929302**	**1.830103263**	**2.473553335**
**SEM**	**0.173225281**	**0.236264271**	**0.30339492**	**0.443865239**	**0.599924804**

veh/OVA	PBS	6.25 mg/ml	12.5mg/ml	25 mg/ml	50 mg/ml
1	2.786801	4.245464	6.553367	6.845941	7.809539
2	1.970389	3.923211	4.348962	4.213654	9.601665
3	1.669809	1.884261	2.420316	3.590176	7.965455
4	1.333673503	1.391791817	4.683917645	5.190429891	7.480905011
5	3.193266894	3.86587285	4.442821423	5.633975321	8.962437019
6	2.701012942	3.436848828	4.400998824	6.793525286	8.492564913
7	1.238656788	1.562640568	2.302457763	2.532308521	3.617796351
8	7.972256896	9.329863936	10.38877803	11.3575847	11.70565069
9	2.095959616	3.039351797	4.846876914	8.105066331	8.121421045
10	1.432331961	2.227089729	2.85718111	5.845535692	6.327994486
11	1.461740036	1.704038151	2.033571442	2.865562987	3.735982847
12	2.158738473	3.420521752	3.810083501	3.678285748	3.533098095
13	1.483194032	3.053558113	3.462646565	6.795509253	5.164676183
14	2.865586853	7.928479366	7.96158687	9.812913305	10.9329385
15	1.404442	2.460712	4.879361	6.362518	10.17541
16	3.187123	3.420627	5.313719	6.896834	7.051217
17	2.108874	4.160121	7.06501	10.37126	14.30859
18	1.434063	2.513598	3.000785	4.825466	3.615096
19	1.884925	2.399977	4.560665	5.430389	6.81736
**Average**	**2.335939158**	**3.472001469**	**4.701742373**	**6.16562816**	**7.653673534**
**STDEV**	**1.508559656**	**2.03078887**	**2.100508968**	**2.448688165**	**2.965930036**
**SEM**	**0.34608732**	**0.465894919**	**0.481889806**	**0.561767592**	**0.680431016**

ANG (0.3mg/kg)	PBS	6.25 mg/ml	12.5 mg/ml	25 mg/ml	50 mg/ml
1	2.145703926	4.502792196	5.63371692	5.505112095	7.413176721
2	1.222753024	1.528391965	2.416176886	4.480043065	5.843548205
3	1.064974358	1.949882358	1.024482379	3.060070388	4.075619596
4	1.051917884	1.482286692	1.944390011	3.236036874	3.858982058
5	1.350430953	1.71268717	3.784200575	3.512157507	3.30809756
6	3.186782729	2.939160825	2.400344404	3.213053857	2.631425574
7	1.363143884	2.317486566	2.767331416	4.936880849	7.216570384
8	2.10297123	1.933733338	3.583633438	2.809195638	2.170945182
9	1.717499491	3.69347949	5.812109346	5.703277617	6.205803977
10	0.919094083	0.969724763	2.924190546	4.354521459	10.25515009
11	1.419780706	2.7904292	4.500558774	8.443662214	8.562212331
12	1.756345	3.138458	3.594109	3.902178	3.949891
13	1.540489	2.609356	2.052349	2.857839	5.48689
14	1.785418	1.915873	2.245679	3.516507	2.716295
15	1.574655	2.303975	2.568068	2.985574	9.330479
16	1.867421	2.342235	4.48949	6.278083	11.4665
**Average**	**1.629336267**	**2.383121973**	**3.233801856**	**4.299637035**	**5.905724167**
**STDEV**	**0.550555426**	**0.891301887**	**1.346094107**	**1.553898138**	**2.896424493**
**SEM**	**0.137638856**	**0.222825472**	**0.336523527**	**0.388474535**	**0.724106123**

A779(1mg/kg)	PBS	6.25 mg/ml	12.5 mg/ml	25 mg/ml	50 mg/ml
1	1.632276	1.728674	3.11748	4.337849	7.048918
2	2.834502	3.102141	5.172264	10.63974	11.18731
3	1.206869161	2.33773353	3.157959661	4.569951938	7.847254355
4	4.59768	7.51057	6.712295	7.601	8.099102
5	2.787687	4.08055	4.265786	4.608402	4.885869
6	4.115658	3.902595	5.043948	4.818745	3.445503
7	3.580681	3.899987	5.726535	5.907108	7.506005
8	1.880699471	3.249450116	5.030056394	7.140554959	8.488084933
9	2.41174133	4.07492068	4.932462502	7.403053894	12.13946164
10	1.997134236	2.991280402	3.451355537	4.386132545	5.920854301
11	2.428909825	2.728497089	5.20775589	4.934228185	6.159706284
12	2.587328831	5.521178579	8.203540198	9.776532129	7.011641979
13	1.889952	1.937344	2.715798	4.844737	4.817058
14	2.966425	4.745305	6.319416	8.343743	10.7177
15	2.18376	4.386659	5.899426	7.683186	7.49785
16	2.026238	2.000686	3.53324	5.847313	8.862031
**Average**	**2.570471366**	**3.637348212**	**4.905582386**	**6.427642291**	**7.602146844**
**STDEV**	**0.905165095**	**1.493273139**	**1.493390528**	**2.004476871**	**2.363914272**
**SEM**	**0.226291274**	**0.373318285**	**0.373347632**	**0.501119218**	**0.590978568**

DEX	PBS	6.25 mg/ml	12.5 mg/ml	25 mg/ml	50 mg/ml
1	0.889240268	1.38960125	2.882953914	2.943685071	4.712056001
2	1.236093179	5.639046098	6.971641457	6.183168068	5.837174578
3	1.141002934	2.161930135	2.78990764	6.543448236	7.024951091
4	2.318152091	2.906866222	3.422263293	5.516587879	5.603398188
5	1.305375014	1.415124033	1.434006224	3.538261526	3.039061168
6	2.464317	3.284849	2.466273	4.445787	5.792795
7	2.930020868	5.593082498	2.751506058	3.139429859	10.66228983
8	1.769385378	1.811064587	1.820904339	3.819584939	6.59890646
9	2.448460868	3.587116696	3.856262528	4.464112164	4.74036019
10	1.623331232	3.147974223	4.837305851	4.292387571	5.596021942
11	1.591113	2.537547	3.978387	4.114972	3.952059
12	1.901299	2.304181	2.88177	3.253236	3.75292
**Average**	**1.801482569**	**2.981531895**	**3.341098442**	**4.354555026**	**5.609332787**
**STDEV**	**0.626655663**	**1.416145052**	**1.473499778**	**1.175488495**	**1.976239054**
**SEM**	**0.180899908**	**0.408805863**	**0.425362747**	**0.3393343**	**0.570491075**
