# Supplementary material for: Ang-(1-7)/ MAS1 receptor axis inhibits allergic airway inflammation via blockade of Src-mediated EGFR transactivation in a murine model of asthma
Source: PLoS One. 2019 Nov 1;14(11):e0224163. doi: 10.1371/journal.pone.0224163 (PMC6824568; doi:10.1371/journal.pone.0224163)
Supplement: S8 Table — (PDF) [file pone.0224163.s012.pdf]

S8 Table: Eosinophil chemotaxis data for the different groups

	PBS	OVA	100	1000	A779
	6.3	20.55	7.125	4.575	11.25
	4.8	18.075	9	5.028	12.5
	3.225	21.975	7.575	3.225	9.95
	3.225	9.6	4.275	2.85	12.5
	3.375	12.45	4.1625	2.588	10.25
	2.85	13.55		4.55	
	2.2	15.45		5.95	
		11.75			
